# MHC class II molecules on pancreatic cancer cells indicate a potential for neo-antigen-based immunotherapy

**DOI:** 10.1080/2162402X.2022.2080329

**Published:** 2022-05-27

**Authors:** Renato B. Baleeiro, Christian J. Bouwens, Peng Liu, Carmela Di Gioia, Louisa S. Chard Dunmall, Ai Nagano, Rathistevy Gangeswaran, Claude Chelala, Hemant M. Kocher, Nicholas R. Lemoine, Yaohe Wang

**Affiliations:** a Centre for Cancer Biomarkers and Biotherapeutics, Barts Cancer Institute, Queen Mary University of London, London, UK; b Centre for Tumour Biology, Barts Cancer Institute, Queen Mary University of London, London, UK; c Research Centre for Molecular Oncology, National Centre for International Research in Cell and Gene Therapy, School of Basic Medical Sciences, Academy of Medical Sciences, Zhengzhou UniversitySino-British, Zhengzhou, Henan, China

**Keywords:** Neo-antigens, cancer immunotherapy, MHC-II-positive tumors, cytotoxic CD4+ T-cells, pancreatic cancer

## Abstract

MHC class II expression is a hallmark of professional antigen-presenting cells and key to the induction of CD4+ T helper cells. We found that these molecules are ectopically expressed on tumor cells in a large proportion of patients with pancreatic ductal adenocarcinoma (PDAC) and on several PDAC cell lines. In contrast to the previous reports that tumoral expression of MHC-II in melanoma enabled tumor cells to evade immunosurveillance, the expression of MHC-II on PDAC cells neither protected cancer cells from Fas-mediated cell death nor caused T-cell suppression by engagement with its ligand LAG-3 on activated T-cells. In fact and surprisingly, the MHC-II/LAG-3 pathway contributed to CD4+ and CD8+ T-cell cytotoxicity toward MHC-II-positive PDAC cells. By combining bioinformatic tools and cell-based assays, we identified a number of immunogenic neo-antigens that can be presented by the patients’ HLA class II alleles. Furthermore, CD4+ T-cells stimulated with neo-antigens were capable of recognizing and killing a human PDAC cell line expressing the mutated genes. To expand this approach to a larger number of PDAC patients, we show that co-treatment with IFN-γ and/or MEK/HDAC inhibitors induced tumoral MHC-II expression on MHC-II-negative tumors that are IFN-γ-resistant. Taken together, our data point to the possibility of harnessing MHC-II expression on PDAC cells for neo-antigen-based immunotherapy.

## Introduction

Pancreatic ductal adenocarcinoma (PDAC) is one of the most aggressive tumor types, with an extremely poor prognosis. Without active treatment, patients with metastatic PDAC have a mean survival of 3–5 months.^1^ Advances in surgical and adjuvant treatments have only marginally improved overall survival rates since the 1970s. Therefore, novel therapeutic approaches are urgently needed.

Recently, attention in cancer therapy has focused more heavily on immune-based strategies as these therapies act through a mechanism that is distinct from chemotherapy and radiotherapy and represent a non-cross-resistant treatment option.^2^ Immune-based therapies aim to stimulate robust T-cell responses against tumor antigens, but significant challenges exist in the development of these regimes. These include poor immunogenicity of the tumors and the presence of a highly immunosuppressive environment within the tumor.^3,4^ The clinical potential of various tumor vaccination strategies has been demonstrated in early phase clinical trials, with some promising immunological and clinical responses in PDAC patients.^5–8^ However, a number of hurdles still exist in the development of an effective immunotherapy for PDAC. Crucially, immune cell subpopulations and specific tumor antigens must be identified that elicit a strong and specific immune response, as failure of past cancer vaccine trials can be attributed in large part to the cell types targeted and selection of inappropriate tumor antigens that have weak inherent immune potential.^9,10^ Additionally, the failure of the immune system to eradicate PDAC may be due to the loss of immunogenicity caused by abnormal expression of major histocompatibility complex (MHC) molecules.^11–13^

The MHC genes encode two major classes of polymorphic proteins, represented by MHC class I (human leukocyte antigen (HLA)-A, B and C) and MHC class II molecules (HLA-DR, DP and DQ). MHC class I proteins are expressed in virtually all nucleated cells, whereas MHC class II expression is restricted to professional antigen-presenting cells (APCs), which include dendritic cells (DCs), B lymphocytes and monocytes/macrophages.^14–16^ While MHC class I molecules bind endogenous processed peptides to present to CD8+ T-cells, MHC class II molecules bind exogenous peptides and present them to the antigen-specific CD4+ T helper (Th) lymphocytes.

Interestingly, ectopic MHC class II expression was observed on a number of human tumor cells, including breast cancer,^17^ carcinoma of the larynx,^18^ colon carcinoma,^19^ melanoma,^20^ and renal cell carcinoma.^21^ MHC class II expression in these cases may be a result of local secretion of inflammatory cytokines such as IFN-γ and TNF-α by tumor-infiltrating lymphocytes, as MHC-II expression was correlated with more intense CD4+ T-cell infiltration compared with MHC-II-negative tumors. The biological significance of MHC class II expression by tumor cells is controversial and has not yet been clarified. Some studies show an association of MHC-II with a better prognosis in breast, colon and larynx carcinoma,^18,19,22^ while the expression of MHC-II molecules by melanoma cells has been linked with worse prognosis.^23–25^ However, despite poorer prognosis, melanoma patients bearing MHC-II-positive tumors have a better response to checkpoint inhibitors than their MHC-II-negative counterparts.^26,27^

The lymphocyte activation gene-3 (LAG-3) protein, expressed by activated CD4+ and CD8+ T-cells and regulatory T-cells (T reg) is the canonical ligand for MHC-II. LAG-3 is a homolog to CD4 and binds to MHC-II with a higher affinity than CD4.^28^ Engagement of MHC-II with LAG-3 on LAG-3-positive T lymphocytes triggers intracellular signaling ^29,30^ leading to suppression of T-cell proliferation, cytokine production and effector functions.^31–33^ There is also some indication of its involvement in immunosuppressive functions of tumor-infiltrating T-cells in prostate cancer.^34^ Due to its possible role in the downregulation of T-cell responses in cancer, LAG-3 is regarded as an immune checkpoint receptor to be targeted through cancer immunotherapy approaches.^35–37^

Although the expression of MHC class II molecules on human PDAC has been reported in a few studies,^38,39^ its functional role in this malignancy has not been clearly elucidated. Here, we confirmed the expression of MHC-II on PDAC cells in a large proportion of patients and on several human PDAC cell lines. In view of these observations, we set out to investigate the functional role of MHC-II molecules in PDAC cells. Our results shed light on the function of MHC-II molecules on tumor cells and indicate the possibility that MHC-II expression on PDAC cells can be harnessed for immunotherapeutic approaches.

## Results

### Expression of MHC class II on PDAC cells

**Figure 1.**
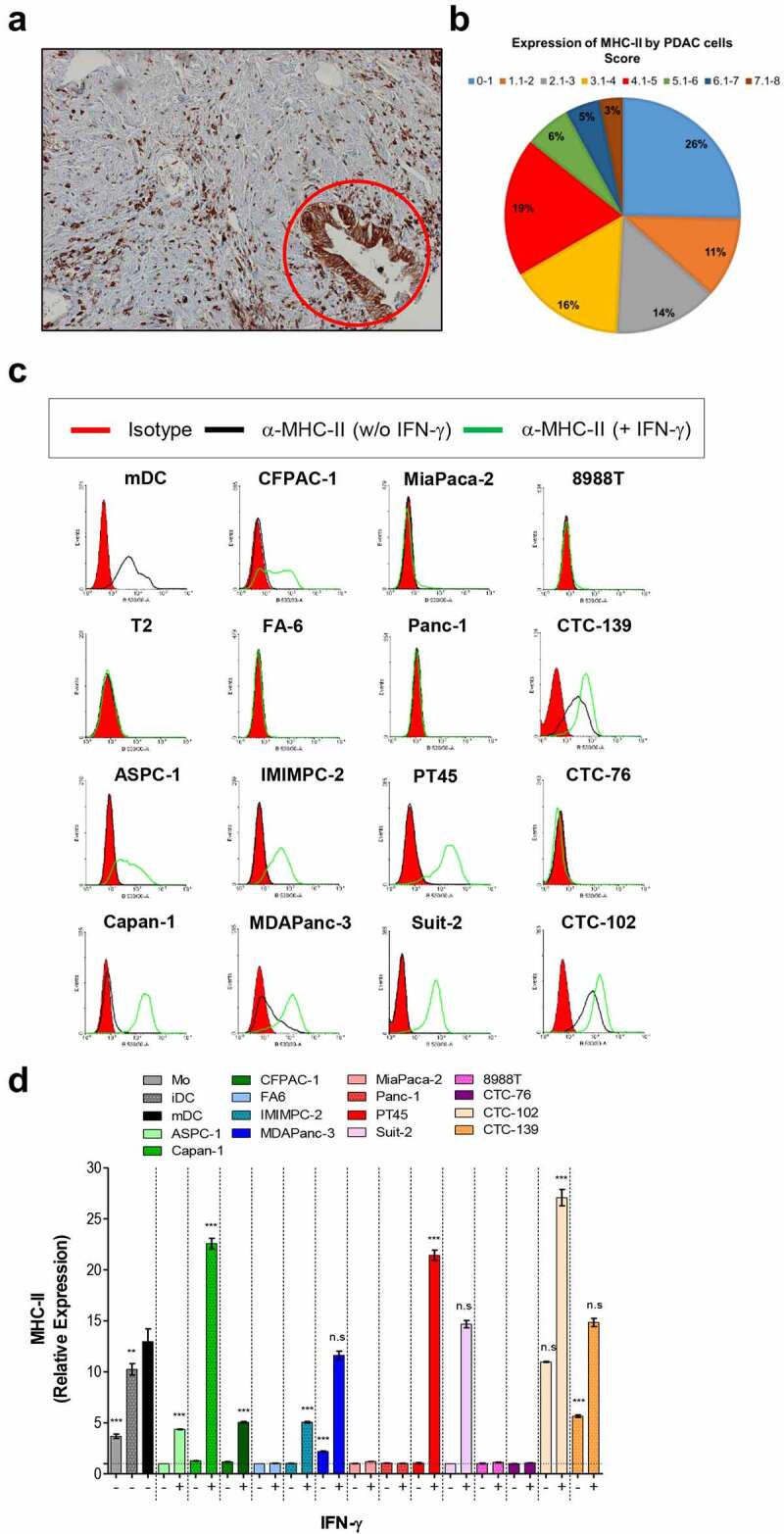
PDAC cells express MHC class II molecules. (a) The expression of MHC-II in paraffin-embedded sections of PDAC from a patient was determined by immunohistochemistry using a specific anti-pan MHC-II antibody as described in Materials and Methods. The red circle indicates MHC-II-positive tumor cells. (**b**) Pie chart showing the percentage of patients and their respective scores for tumoral MHC-II expression. MHC class II expression on untreated or IFN-γ treated primary and circulating PDAC cell lines was determined by flow cytometry. The histograms show the expression of the MHC-II by cells from one representative experiment (**c**), and the graph in (**d**) shows mean fold changes with S.E.M of the expression of the marker from the 3 experiments analyzed. Data in (**d**) were analyzed by one-way ANOVA comparing each MHC-II positive PDAC cell line with mDC. **p < 0.01; ***p < 0.001; n.s: non-significant. mDC and T2 shown in **C** were used as positive and negative controls for MHC-II expression, respectively. Mo, iDC and mDC in the graph in **D** were used as positive controls for MHC-II expression by APCs in different stages of differentiation. Magnification of image shown in (**A**): 10×.

Expression of MHC class II molecules was first assessed using paraffin-embedded tissue from 63 patients with PDAC using immunohistochemistry (IHC). MHC class II expression on tumor cells was observed in 49 out of the 63 PDAC cases (77.7%) (Figure 1(a,b) and Table S1A). Of these, 26 cases revealed scores equal to or above 4, which shows that roughly half of the tumor cells are expressing MHC-II molecules at moderate or high levels (Table S1A). The expression of MHC-II by PDAC cells was associated with immune cell infiltrates (Fig S1), compatible with an inflammatory micro-environment that could induce ectopic expression of MHC-II on nonprofessional APCs such as tumor cells.

Next, we sought to assess expression of MHC-II on human PDAC cell lines (8988 T, ASPC-1, Capan-1, CFPAC-1, FA6, IMIMPC-2, MDAPanc-3, MiaPaca-2, Panc-1, PT45, SUIT-2), and circulating tumor cell lines (CTC-76, CTC-102 and CTC-139) established from cells isolated from the blood of patients with PDAC.^40^ As determined by flow cytometry (FC), three out of all the 14 cell lines analyzed expressed variable levels of cell-surface MHC-II. Following IFN-γ treatment, MHC class II surface expression was upregulated in these cell lines and *de novo* expression was observed in six additional cell lines (Figure 1(c,d)). While spontaneous expression of MHC-II was seen in one out of 11 primary PDAC lines, among the CTC lines two out of the three lines exhibited MHC-II expression. Notably, upon treatment with IFN-γ the cell lines Capan-1, PT45 and CTC-102 expressed higher levels of MHC-II than mature DCs, the main professional antigen-presenting cells (Figure 1(d)).

### Engagement of MHC-II on PDAC cells does not confer protection to fas-mediated cell death

Engagement of tumoral MHC-II with LAG-3 on T-cells was reported to protect melanoma cells from apoptosis.^37^ To ascertain the biological significance of MHC-II expression on PDAC cells, we first analyzed the effect of the engagement of MHC class II molecules on Fas-induced cell death using the PDAC cell line SUIT-2 untreated (MHC II-negative) or treated with IFN-γ (MHC-II-positive). SUIT-2 cells expressed substantial levels of Fas regardless of treatment with IFN-γ and were sensitive to Fas-mediated cell death (Fig S2). SUIT-2 cells were pre-incubated or not with the MHC-II-ligand sLAG-3 and/or the MHC-II agonist antibody L243 for 1 h and were then treated with anti-Fas antibody. Staining of the cells with EthD-1 and calcein-AM indicated that treatment with anti-Fas antibody for 48 h induced cell death in 48% of untreated and in 72% of IFN-γ-treated SUIT-2 cells (Fig S3). Cell death was not decreased in the presence of either sLAG-3, anti-MHC-II antibody or a combination of sLAG-3 and anti-MHC-II in both untreated and IFN-γ-treated cells, indicating that unlike previously reported in melanoma, engagement of MHC-II on PDAC cells does not confer protection against Fas-mediated cell death.

### Inhibition of the MHC-II/LAG-3 axis impairs cytotoxic function of CD8+ T-cells

**Figure 2.**
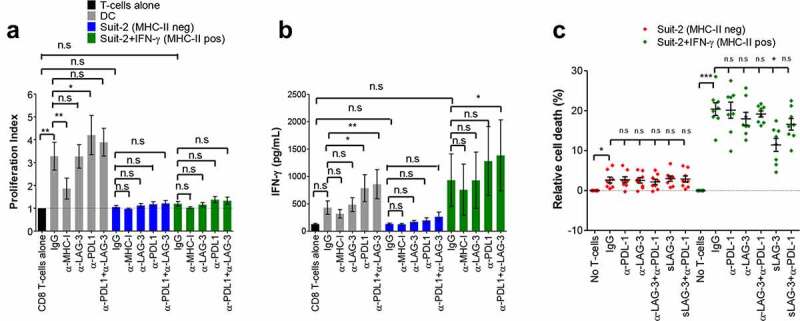
MHC-II/LAG-3 axis contributes to CD8+ T-cells cytotoxic function. Allogenic activated CD8+ T-cells from healthy donors were cultured with MMC-treated PDAC cells (T-cell:tumor ratio 2:1) in the presence of blocking antibodies as indicated or isotype control (IgG) for 4 days. T-cell proliferation was determined by CFSE staining and results are expressed as mean values and S.E.M of the proliferation index (**A**) determined as described in Materials and Methods. The supernatants of the co-cultures were collected and assayed for IFN-γ by ELISA (**B**). Allogenic mature DCs (T-cell:DC ratio 10:1) were used as positive control in these experiments. For determination of cytotoxic activity of CD8+ T-cells, activated CD8+ T-cells from HLA-A2-negative donors were incubated overnight with untreated or IFN-γ-treated HLA-A2-positive Suit-2 cells. Where indicated, blocking antibodies, isotype control or sLAG-3 was added. Cell viability of tumor cells in these co-cultures was determined by staining with EthD-1 and calcein-AM. Tumor cells gated on HLA-A2+ that were negative for EthD-1 and positive for calcein-AM were considered viable cells (**C**). All data were analyzed by one-way ANOVA. *P < 0.05; **p < 0.01; ***p < 0.001; n.s: non-significant. Values shown in A and B were from independent experiments with 3 donors; in C is shown values from 4 donors performed in duplicate.

The MHC-II ligand LAG-3, expressed on activated CD4+ and CD8+ T-cells, is described as a checkpoint molecule, decreasing T-cell effector functions and proliferation upon engagement with MHC-II.^31–37^ To investigate whether MHC-II expressed by PDAC cells can functionally interact with LAG-3, thereby inhibiting T-cell function, we analyzed the effect of the blockade of LAG-3 on the proliferation of LAG-3-positive T-cells as well as the production of IFN-γ by these cells in an allogenic system. As activated but not resting T-cells express LAG-3,^29,30,39^ allogenic PBMCs from healthy donors were activated with anti-CD3/CD28-coated beads plus IL-12 for 5 days followed by a 48 h resting period, and the expression of LAG-3 was observed to increase from ~1.5% to ∼45% of CD8+ T-cells (Fig S4). Untreated SUIT-2 cells (MHCII-negative) did not induce proliferation of CD8+ T-cells (Figure 2(a)) nor production of IFN-γ (Figure 2(b)). Although IFN-γ-treated SUIT-2 cells (MHCII-positive) induced a weak proliferation (Proliferation index = 1.2) as well as some increase in IFN-γ (from 130.5 pg/mL to 931.8 pg/mL, CD8+ T-cells alone vs CD8+ T-cells + IFN-γ-treated Suit-2), the difference did not reach statistical significance. The blockade of LAG-3 did not enhance the MHC-II-positive PDAC cell-induced proliferation of allogenic activated CD8+ T lymphocytes (Figure 2(a)) nor induced production of IFN-γ (Figure 2(b)) by these cells, suggesting that the MHC-II/LAG-3 axis does not play a role in the clonal expansion and IFN-γ production. As IFN-γ-treated PDAC cells express high levels of PD-L1 (Fig S5), a well-characterized checkpoint molecule ligand, we hypothesized that the PDAC cells were inhibiting T-cell function through the PD-1/PD-L1 pathway despite the blockade of LAG-3. We observed that although the dual blockade of PD-L1 and LAG-3 caused a slight boost in proliferation and IFN-γ production by CD8+ T-cells in co-culture with tumor cells, the difference was not greater than the blockade of only PD-L1. Similarly, blockade of PD-L1 or PD-L1 together with LAG-3 led to enhanced T cell proliferation and IFN-γ production in the control co-cultures where the stimulating cells were allogenic mDCs.

Given that the major function of activated CD8+ T-cells is to kill target cells, we next tested whether blocking the MHC-II/LAG-3 axis could enhance T-cell-mediated cytotoxicity. Following overnight co-culture, activated CD8+ T-cells killed an average of 20.4% of the MHC-II-positive SUIT-2 cells (Figure 2(c)). Surprisingly, blockade of the MHC-II/LAG-3 axis decreased the cytotoxicity of the CD8+ T-cells toward the MHC-II-positive SUIT-2 cells, indicating that LAG-3 signaling in CTLs plays an important role in their main effector function. The blocking was more effective when sLAG-3 was used, decreasing the cell death from 20.5% to 11.4%. Anti-LAG-3 antibodies reduced cell death from 20.5% to 18.5%, but the difference did not reach statistical significance. This is probably due to differences in the blocking capacity of these two molecules. Addition of anti-LAG-3 or sLAG-3 into the co-cultures of CTL with MHC-II-negative SUIT-2 cells had no effect on the cytotoxicity against the target cells (Figure 2(c)), indicating that the activity of LAG-3 is mediated by MHC-II. Taken together, these data show that the MHC-II/LAG-3 axis does not promote suppression of activated CD8+ T-cells, but rather plays an important role in their cytotoxic activity.

### MHC-II molecules on PDAC cells seem to be loaded with tumor-derived peptides despite the absence of HLA-DM and poor immunostimulatory capacity

**Figure 3.**
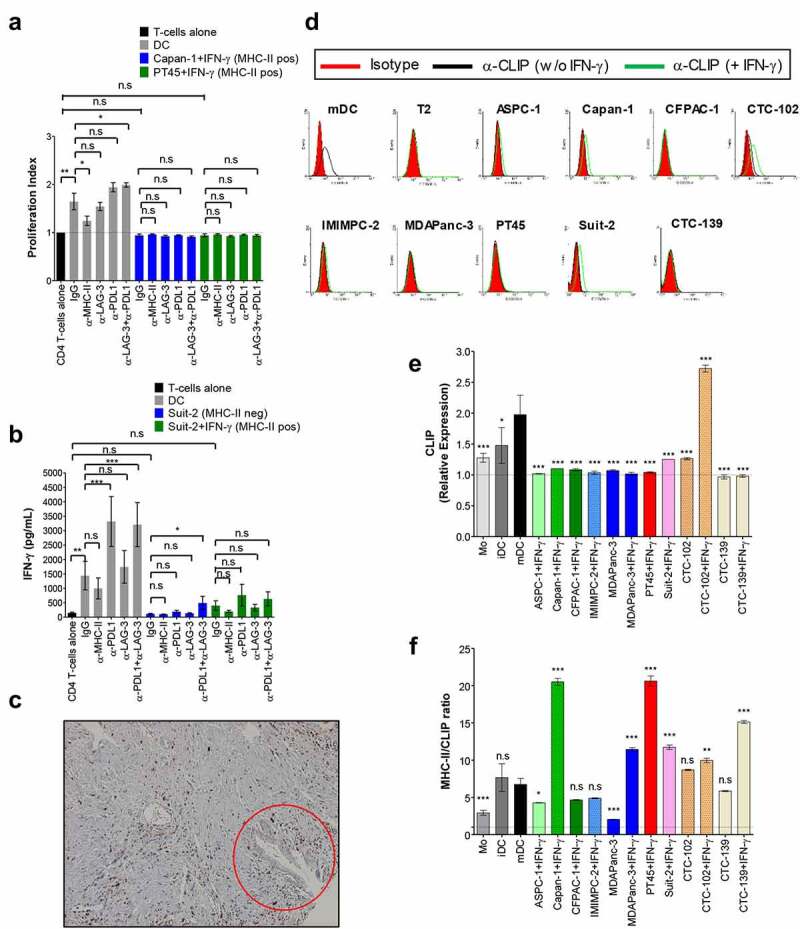
MHC-II on PDAC cells seem to be loaded with tumor-derived peptides despite the absence of HLA-DM and poor immunostimulatory capacity. Allogenic activated CD4+ T-cells were cultured with MMC-treated PDAC cells (T-cell:tumor ratio 2:1) in the presence of blocking antibodies as indicated or isotype control (IgG) for 4 days. T-cell proliferation was determined by CFSE as in Figure 2 and results are expressed as mean values and S.E.M of the proliferation index (**A**). Supernatants of co-cultures of MHC-II-negative and – positive PDAC lines were collected and assayed for IFN-γ by ELISA (**B**). Allogenic mature DCs (T-cell:DC ratio 10:1) were used as positive control. Expression of HLA-DM in PDAC specimens was assessed by IHC (**C**). Tumor cells indicated by the red circle are positive for MHC-II as shown in Figure 1(a). Expression of CLIP on untreated or IFN-γ-treated PDAC cell lines was determined by staining cells with anti-CLIP antibody and analyzed by flow cytometry. The histograms show the expression of CLIP by cells from one representative experiment (**D**), and the graph in (**E**) shows mean fold changes with S.E.M of the expression of the marker from the three experiments combined. In (**F**) is shown the ratio between MHC-II and CLIP. T2 cells and mDC in **D** were used as negative and positive control for CLIP, respectively; Mo, iDC and mDC shown in **E** and **F** were used as positive control for the expression of CLIP. All data were analyzed by one-way ANOVA. *P < 0.05; **p < 0.01; ***p < 0.001; n.s: non-significant. P values shown in **E** and **F** refer to the comparison of the indicated cell line vs. mDC. Values shown in **A** were from independent experiments with 4 donors; and in **B** is shown values from 6 donors. Magnification of image shown in (**C**): 10×.

To ascertain the role of the MHC-II/LAG-3 axis in the function of CD4+ T-cells, allogenic activated CD4+ T-cells from healthy donors were cultured with MHC-II-negative and -positive PDAC cells in the presence of LAG-3 blocking antibody and proliferation and IFN-γ production were assessed. Following the activation of CD4+ T-cells with CD3/CD28-coated beads plus IL-12, the percentage of LAG-3-positive cells increased from ~1.5% to ∼5% of CD4+ T-cells, a more modest increase if compared with the CD8+ T-cells (Fig S4). Blockade of LAG-3 had no significant effect on the proliferation of CD4+ T-cells (Figure 3(a)) or IFN-γ production by these cells (Figure 3(b)) in co-culture with MHC-II-negative or MHC-II-positive tumor cells. In fact, tumor cells were not able to induce proliferation of CD4+ T-cells even when PD-L1 was blocked, which complicates drawing conclusions about the role of the MHC-II/LAG-3 axis in this important T-cell function. However, when mDCs were used as stimulators, they induced both proliferation (Proliferation index = 1.64) and IFN-γ responses (from 148.7 pg/mL to 1443 pg/mL, CD4+ T-cells alone vs CD4+ T-cells + DC), which was raised with inhibition of PD-L1, but remained unchanged upon blockade of LAG-3 (Figure 3(a,b)).

The fact that PDAC cells expressing MHC-II did not induce CD4+ T-cell proliferation and only modest IFN-γ responses even upon blockade of PD-L1 made us question if PDAC cells display functional MHC-II molecules on their surfaces. A key intracellular event in antigen processing for presentation on MHC-II is the displacement of the invariant chain peptide CLIP from the binding groove of MHC-II by HLA-DM for binding of tumor-derived peptides. The analysis of HLA-DM on PDAC specimens revealed expression by infiltrating immune cells but not by PDAC cells (Figure 3(c) and Table S1B). The absence of HLA-DM in MHC-II-positive cells might lead to the presentation of non-immunogenic CLIP instead of potentially immunogenic peptides by tumor cells, which could explain their poor immunostimulatory capacity in the allogenic T-cell assays. However, MHC-II positive PDAC cell lines exhibited very low expression of cell surface CLIP (Figure 3(d,e)) as well as a high ratio of MHC-II/CLIP (Figure 3(f)), indicating that most MHC-II molecules are loaded with antigenic peptides, despite the lack of HLA-DM. Given the fact that mDCs expressing lower levels of MHC-II and lower ratio of MHC-II/CLIP than the PDAC cells used in these experiments (Figure 1(c-f)) were potent immunostimulatory cells, the poor stimulatory capacity of PDAC cells may be attributed to the lack of co-stimulatory molecules (Fig S6). Altogether, our data suggest that a substantial proportion of MHC-II molecules expressed on the surface of PDAC cells are loaded with tumor-derived peptides and therefore have the potential to serve as functional antigen-presenting molecules.

### Activated CD4+ T-cells kill MHC-II-positive PDAC cells

**Figure 4.**
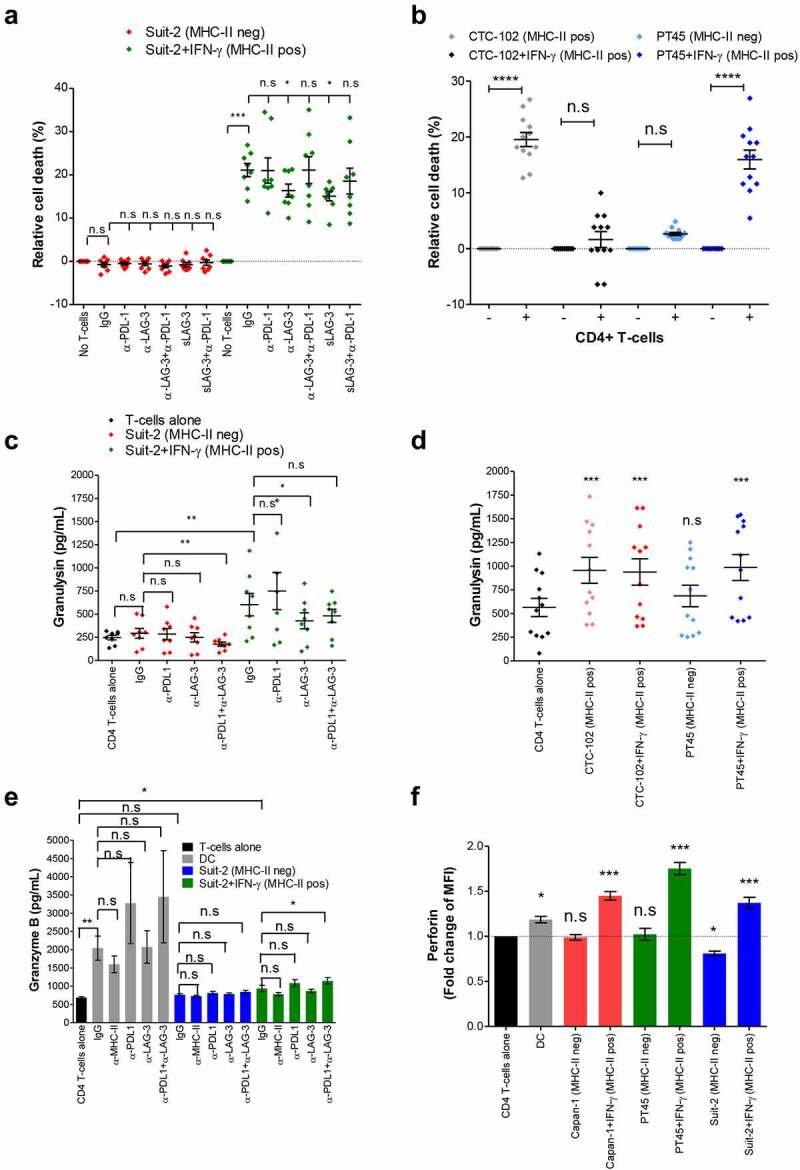
Activated CD4+ T-cells acquire cytotoxic function and kill MHC-II positive PDAC cells. Allogenic activated CD4+ T-cells from healthy donors were incubated overnight with PDAC cells (T-cell:tumor ratio 2:1). Where indicated, the cultures were treated with blocking antibodies or isotype control (IgG), or soluble LAG-3. Tumor cell viability was determined by EthD-1 and calcein-AM staining (**A** and **B**). The supernatants of the co-cultures were collected and assayed for granulysin (**C** and **D**) by ELISA. Granzyme B was detected in the supernatant of 4-day co-cultures of CD4+ T-cells and tumor by ELISA (**E**) and presence of intracellular perforin in CD4+ T-cells from these co-cultures was determined by flow cytometry (**F**). Allogenic mature DCs (T-cell:DC ratio 10:1) were used as stimulator of CD4+ T-cells as positive control for granzyme B release (**E**) and perforin expression (**F**). The data were analyzed by one-way ANOVA (**A, C, D, E** and **F**) or two tailed paired t test (**B**). *P < 0.05; **p < 0.01; ***p < 0.001; n.s: non-significant. P values shown in **D** and **F** refers to the comparison of the indicated cell line vs. CD4+ T-cells alone. Values shown in **A** and **C** were from independent experiments with 4 donors performed in duplicate; in **B** and **D** are shown values from 6 donors performed in duplicate; in **E** and **F** values represent independent experiments from 6 and 4 donors, respectively.

Some reports show that CD4+ T-cells can differentiate into cytotoxic cells expressing granzymes and Fas Ligand and directly kill tumor cells expressing MHC-II.^41–44^ To ascertain the role of CD4+ T-cells in the direct targeting of MHC-II-positive PDAC cells, we co-cultured purified activated CD4+ T-cells with IFN-γ-stimulated PDAC cells overnight and assessed cytotoxicity. Activated CD4+ T-cells were able to kill an average of 21.1% of MHC-II-positive SUIT-2 cells but showed no cytotoxicity toward MHC-II-negative SUIT-2 cells (Figure 4(a)). Blockade of the MHC-II/LAG-3 axis with either anti-LAG-3 antibody or sLAG-3 significantly reduced cytotoxicity to 16.35% and 15% of MHC-II-positive SUIT-2 cells, respectively, pointing to an important role of LAG-3 signaling in the effector function of CD4+ T-cells, similar to what we had observed for CD8+ T-cells. Another two PDAC cell lines CTC-102 and PT45 became targets for CD4+ T-cells when they expressed MHC-II (Figure 4(b)). While activated CD8+ T-cells were very effective at killing MHC-II-negative PT45 cells (Fig S7), CD4+ T-cells in the same experimental setting failed to do so, confirming once again that CD4+ T-cells exert cytotoxic activity in an MHC-II-dependent manner. It is worth noting that while the MHC-II positive CTC-102 cell line proved to be an efficient target for the activated CD4+ T-cells, the treatment of these cells with IFN-γ rendered them resistant to CD4+ T-cell-mediated cytotoxicity, despite the increase in MHC-II expression. IFN-γ-treated CTC-102 was also resistant to MHC-I-dependent and CD8+ T-cell-induced cell death (Fig S7), which suggests that these cells may have acquired a mechanism that protects them against cytolytic molecules used by both CD4+ and CD8+ T-cells. The mechanism by which activated CD4+ T-cells kill MHC-II positive PDAC cells is probably via secretion of cytotoxic granules, as increased amounts of granulysin (Figure 4(c,d)) and granzyme B (Figure 4(e)) were found in the supernatant and intracellular expression of perforin (Figure 4(f) and Fig S8) was increased when they were incubated with MHC-II-positive, but not MHC-II-negative PDAC cell lines. It is worth noting that the inhibition of LAG-3 with anti-LAG-3 antibody caused a significant decrease in the release of granulysin (Figure 4(c)), in agreement with the role of the MHC-II/LAG-3 axis in the cytotoxic function of these cells. Importantly, there is a trend for lower granzyme B secretion when the co-cultures contained anti-MHC-II-specific antibodies (Figure 4(e)), indicating that CD4+ T-cells are capable of recognizing MHC-II-expressing PDAC cells via their T-cell receptor. Altogether, these results show that activated CD4+ T-cells can become cytotoxic and kill MHC-II-positive PDAC cells as efficiently as CD8+ T-cells, with LAG-3 contributing to their cytotoxic activity.

### PDAC patients’ tumors can generate MHC class II neo-epitopes

**Figure 5.**
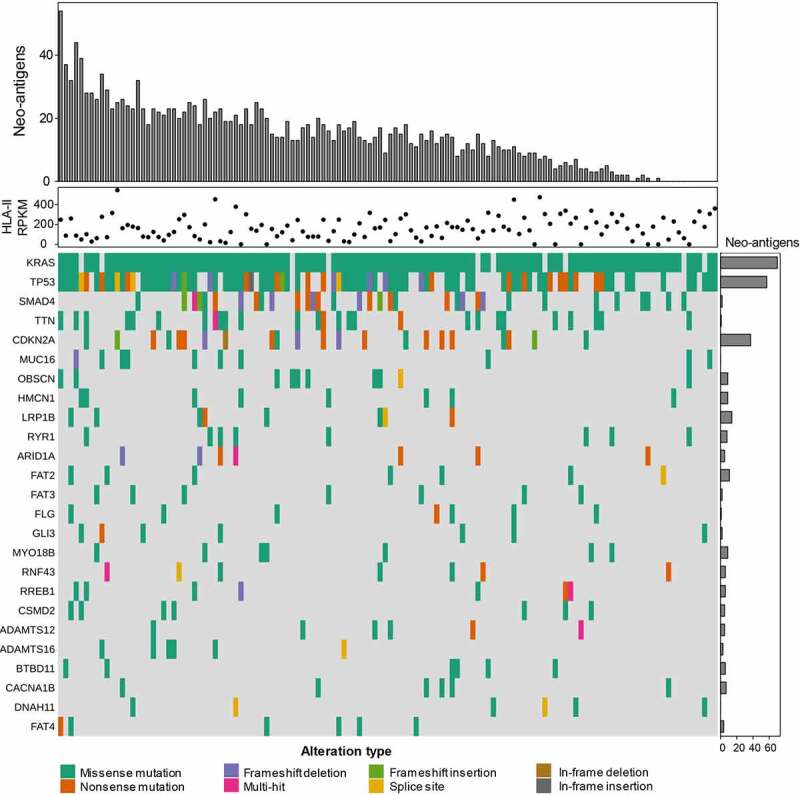
PDAC patients can generate MHC class II neo-epitopes. Using exome and transcriptome data from 127 PDAC patients, we identified neo-antigens that can be presented by the patients’ HLA class II alleles. Each column is tied to one patient over the entire plot. The top barplot shows the total number of predicted neo-epitopes per patient. These are restricted to each patients HLA-II alleles and combinations thereof. The dot plot graph shows the expression of HLA-II as determined by seq2HLA. The oncoplot in the bottom represents the top 25 genes (rows) mutated per patient (column). The barplot annotation on the right represents the frequency these genes occur as HLA-II restricted neo-antigens in the dataset.

As MHC-II is expressed by tumor cells in a large proportion of patients, and given the fact that when properly activated, CD4+ T-cells can kill MHC-II positive PDAC cells, we reasoned that MHC-II molecules on PDAC cells could be harnessed for neo-antigen-based immunotherapy. To this end, we analyzed the available exome and transcriptome data from 127 PDAC patients and using *in silico* prediction tools, we identified neo-antigens that can be presented by the patients’ HLA class II alleles. Through that analysis, we selected the top 25 mutations (Fig S9) across the patients and identified 14.32 neo-antigens on average with a median of 14 neo-antigens per patient (Figure 5). The number of predicted neo-antigens varied from zero in 11 patients to over 50 in one patient. Interestingly, the number of neo-antigens did not correlate with the expression level of HLA class II molecules, as patients expressing high levels of mRNA for HLA-II molecules did not always generate a high number of neo-antigens and *vice-versa*. Most of the predicted neo-antigens were derived from mutated KRAS and TP53, the top two mutated proteins in the PDAC patients analyzed. Altogether, these data show that PDAC patients can potentially generate a significant number of MHC-II neo-epitopes that could be targeted in immunotherapy strategies.

### In vitro validation is required to predict immunogenicity of MHC-II epitopes identified through bioinformatic prediction algorithms

**Figure 6.**
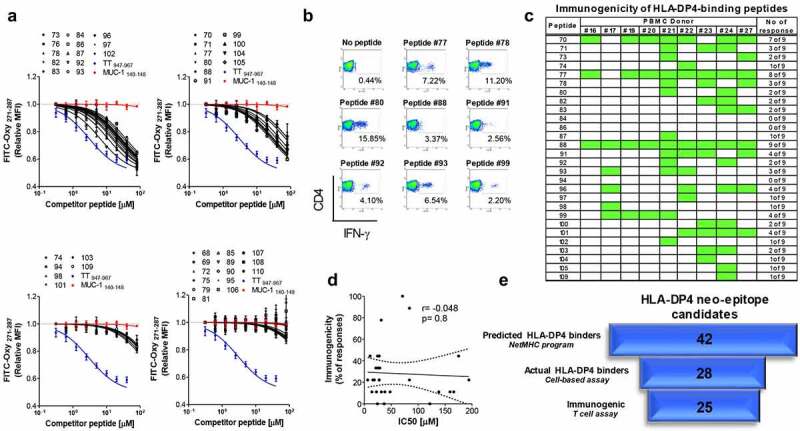
Screening of MHC-II neo-epitopes candidate for binding capacity and immunogenicity. (A) Forty-two peptides representing neo-antigens with good predicted binding affinity to HLA-DP4 molecules were examined in a competitive binding assay using T2 cells transfected with HLA-DP4 (T2-DP4) as described in material and methods. n = 3 per peptide. S.E.M is shown. MUC-1_140–148_ and TT_947–967_ peptides were used as negative and positive control respectively; TT: Tetanus toxin. (**B**) Twenty eight out of the 42 tested peptides showed HLA-DP4-binding capacity and were examined for their immunogenicity in T-cell assays using PBMCs from 9 donors positive for HLA-DP4. CD4+ T-cells isolated from healthy donor blood were stimulated three times with autologous mature dendritic cells pulsed with each of the 28 HLA-DP4-binding peptides. Seven days after the last round of stimulation, CD4+ T-cells were challenged with T2-DP4 cells pulsed with the same peptides or left without peptide. Intracellular IFN-γ was measured by FACS. The dot plots in (**B**) depict eight mutant peptides that elicited strong CD4+ T-cell responses from one representative donors and in (**C**) is shown the response of all donors; green represents the responses. (**D**) Correlation between binding capacity of the peptides as per the IC50 values from cell-based binding assays and immunogenicity, expressed as the percentage of donors responding to each individual peptide. (**E**) Flowchart for the number of mutated peptides identified by whole exome and predicted to bind HLA-DP4 (NetMHC), narrowed down by their capacity to bind HLA-DP4 in cell-based binding assay and the number of peptides validated as actual immunogenic peptides.

Given the large number of neo-epitopes predicted to bind to the patients’ HLA class II alleles in our *in silico* analysis, we asked how many of the predicted peptides are true binders and capable of eliciting CD4+ T-cell responses. To address these questions, we set up a platform to screen the peptides for true binding capacity and immunogenicity. We chose to validate our platform on peptides predicted to bind to HLA-DP4, which is the most widely expressed HLA class II allele worldwide, present in about 70% of the population.^45^ We used available exome data from 100 PDAC patients ^46^ and through *NetMHCIIpan-3.0*, we identified 42 neo-antigens that are predicted to be presented by HLA-DP4 (Table S2). Peptides corresponding to the 42 candidate neo-epitopes were synthesized and tested for their binding capacity to HLA-DP4 in a cell-based competitive binding assay using T2 cells transfected with HLA-DP4. Of the 42 peptides tested, 28 (66.6%) bound to HLA-DP4 with varying degrees of efficiency (Table S3 and Figure 6(a-e)). Peptides 68 and 69 that were predicted to bind to HLA-DP4 with high affinity, ranking in the 0.29 and 0.24 percentile, respectively, turned out not to bind. On the other hand, peptide 96, predicted to be a weak binder, ranking in the 15.9 percentile in terms of affinity, was revealed to be a good binder, ranking in the top five binders in the cell-based binding assay (Table S3-5). To evaluate the immunogenicity of the candidate neo-epitopes *in vitro*, mature DCs were pulsed with individual epitopes and used to stimulate autologous CD4+ T-cells in three weekly stimulation rounds. Seven days after the third stimulation, CD4+ T-cells were challenged overnight with T2-DP4 pulsed with the epitope and intracellular IFN-γ production was used as a measure of T-cell response. We observed that of the 28 epitopes that were confirmed to bind to HLA-DP4, 25 were capable of eliciting CD4+ T-cell responses (Figure 6(b-e)). Interestingly, immunogenicity varied greatly among the epitopes, while peptides 74, 87, 97, 98, 104, 105 and 109 induced responses in only one out of nine donors, peptide 88 was the most immunogenic neo-epitope candidate eliciting CD4+ T-cell responses in all nine donors tested (Figure 6(c)). Even though for any given peptide to induce T-cell response, it must be able to bind to HLA, the affinity to which it binds is not a good predictor of immunogenicity, as there was no correlation between the binding capacity of the peptides to the HLA and immunogenicity in our assays (Table S4 and S5 and Figure 6(d)). In conclusion, of the 42 peptides predicted by bioinformatics tools, only 25 hold the potential to translate into an effective CD4+ T-cell-based immune response (Figure 6(c-e)). These results highlight the need for screening of neo-epitope candidates in cell-based assays and the need for improvement of the current prediction algorithms.

### Human PDAC Capan-1 cell line presents tumor neo-antigens that can be targeted by CD4+ T-cells

**Figure 7.**
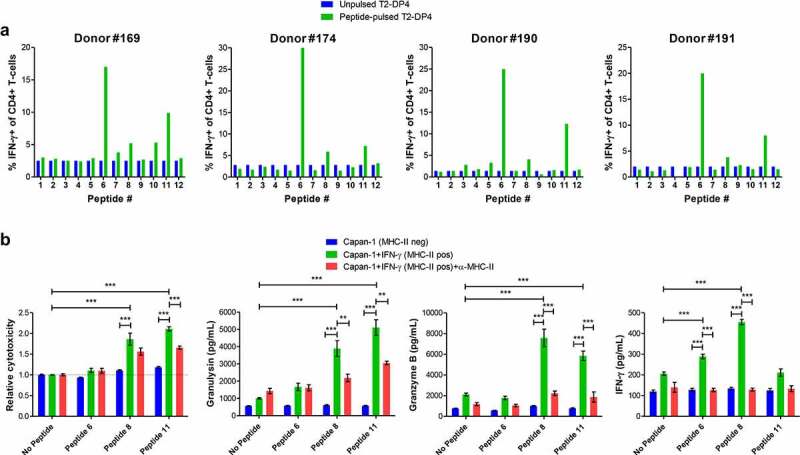
Human PDAC Capan-1 cell line generates immunogenic neo-antigens and can be targeted by CD4+ T-cells. (A) Twelve HLA-DP4-binding peptides were examined for their ability to promote immune responses in immunogenicity T-cell assays using healthy blood samples from 4 donors positive for HLA-DP4. CD4+ T-cells isolated from healthy donor blood were stimulated three times with autologous mature dendritic cells pulsed with each of the 12 HLA-DP4-binding peptides. Seven days after the last round of stimulation, CD4+ T-cells were challenged with T2-DP4 cells pulsed with the same peptides or left without peptide and intracellular IFN-γ was measured by FACS. (**B**) CD4+ T-cells from the donor #190 were stimulated with peptides 6, 8 and 11 as in (**A**). Seven days after the third stimulation they were incubated overnight with MHC-II-negative or MHC-II-positive Capan-1 cells with or without blocking anti-MHC-II antibodies. Supernatants were collected and assayed for LDH (for cytotoxicity), granulysin, granzyme B and IFN-γ. Data shown in (**B**) are from at least three independent repeats. The data were analyzed by one-way ANOVA; **p < 0.01; ***p < 0.001. Graphs show mean ± SEM.

We next sought to investigate whether human PDAC cells can generate immunogenic tumor neo-antigens to be recognized by peptide-specific CD4+ T-cells. For that, we used exome and transcriptome data from Capan-1 cells and selected 12 HLA-DP4-binding neo-antigen peptides (Table S6). Three out of the 12 peptides assessed were immunogenic to the four donors tested (Figure 7(a)). All three immunogenic peptides have been shown to bind to HLA-DP4 in cell-binding assays (Fig S10 and Table S7). CD4+ T-cells from the donor #190 were further stimulated with each of the three immunogenic peptides and challenged with Capan-1 cells. Peptide-stimulated CD4+ T-cells reacted to MHC-II-positive, but not MHC-II-negative Capan-1 cells. Intriguingly, the three peptides seem to induce CD4+ T-cells with distinct functional capabilities; CD4+ T-cells specific for peptide 6 produced IFN-γ, but did not kill Capan-1 cells, while CD4+ T-cells reactive to peptide 11 exhibited killing activity, but produced low IFN-γ. CD4+ T-cells specific for peptide 8 produced high IFN-γ and displayed killing activity. Importantly, killing activity was always followed by the release of high amounts of granulysin and granzyme B (Figure 7(b)). Control CD4+ T-cells cultured the same way, but without peptides, produced low amounts of IFN-γ, granulysin and granzyme B and displayed poor cytotoxic activity against MHC-II-positive Capan-1 cells, which shows that the peptide-specific T cells were recognizing the tumor cells in a specific manner. Collectively, these data indicate that PDAC cells can generate immunogenic neo-antigens that can be used as targets for a CD4+ T-cell-based immunotherapy.

### MEK/ERK and histone deacetylase inhibitors induce MHC-II expression on IFN-γ-resistant PDAC cells

**Figure 8.**
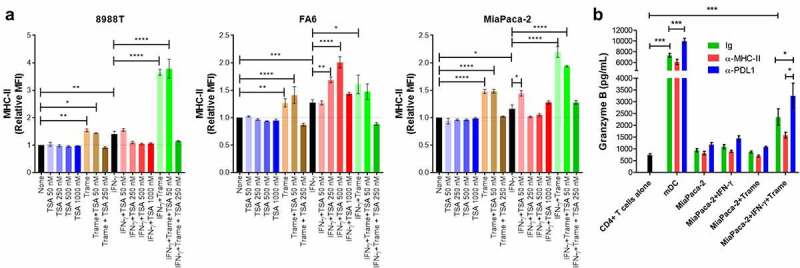
Trametinib and TSA enhance responsiveness to IFN-γ in PDAC cells. (A) 8988 T, FA6 and MiaPaca-2 cells were treated with IFN-γ or left untreated, in the presence or absence of the MEK inhibitor, trametinib and/or histone deacetylase inhibitor TSA at the indicated concentrations for 48 hours. The cells were then collected, stained with anti-MHC-II antibodies and analyzed by flow cytometry. All flow cytometry data were gated on viable, singlets and MFI values for MHC-II were normalized to the untreated cells (none). Data are from three independent experiments. (**B**) Allogenic activated CD4+ T-cells from 4 healthy donors were incubated overnight with MiaPaca-2 cells (T-cell:tumor ratio 5:1). Where indicated, the cultures were treated with blocking antibodies or isotype control (IgG). The supernatants of the co-cultures were collected and assayed for granzyme B by ELISA. Allogenic mature DCs (T-cell:DC ratio 10:1) were used as stimulator of CD4+ T-cells as positive control for granzyme B release. All data were analyzed by one-way ANOVA; * P < 0.05; ** p < 0.01; *** p < 0.001; **** p < 0.0001. Graphs show mean ± SEM.

To be killed by CD4+ T-cells, tumor cells must express MHC-II on their surface. Although MHC-II molecules can be induced by IFN-γ in most PDAC cell lines, some of them fail to express MHC-II even after treatment with IFN-γ (Figure 1). It has recently been shown that treatment of lung cancer cell lines with MEK inhibitors and histone deacetylase (HDAC) inhibitors restores responsiveness of those cells to IFN-γ treatment and induces *de novo* MHC-II expression.^47^ To determine whether blockade of MEK and HDAC pathways can induce expression of surface MHC-II in PDAC cell lines after IFN-γ treatment, we treated the IFN-γ-resistant 8988 T, FA6 and MiaPaca-2 cells with IFN-γ in the presence or absence of MEK inhibitor trametinib and/or HDAC inhibitor TSA for 48 h. IFN-γ treatment induced low expression of MHC-II on the three cell lines. While TSA alone did not induce expression of MHC-II in any of the three cell lines, trametinib alone induced a slight increase in the expression of MHC-II on the three cell lines. Simultaneous treatment of cells with IFN-γ and trametinib increased MHC-II expression compared with IFN-γ alone. Treatment of cells with IFN-γ in the presence of TSA enhanced MHC-II expression in FA6 cells, but not in 8988 T and MiaPaca-2 cells. Furthermore, the combination of both inhibitors did not increase MHC-II expression beyond the levels observed upon treatment with only one inhibitor (Figure 8(a)). More importantly, the expression of MHC-II by MiaPaca-2 cells induced by IFN-γ in conjunction with trametinib rendered these cells susceptible to CD4+ T-cells recognition (Figure 8(b)). Altogether, these results show that MHC-II can be induced in tumor cells resistant to IFN-γ by co-treatment with inhibitors, which could ultimately turn MHC-II-negative into MHC-II-positive tumors and, therefore, potentially targetable by CD4+ T cell therapy.

## Discussion

The main goal of this study was to elucidate the role of MHC class II molecules on PDAC cells. We show that activated CD4+ T-cells acquire cytotoxic features and kill MHC-II-positive PDAC cells as efficiently as CD8+ T-cells. Furthermore, our results suggest that the expression of MHC class II molecules by pancreatic cancer cells can be harnessed for neo-antigen-based immunotherapy. The ability to induce cell death of MHC-II-positive tumors by cytotoxic CD4+ T-cells was previously documented in a murine model of MHC-II-positive melanoma.^48^ However, the findings of that study have not been developed further into a therapy to engage CD4+ T-cells to destroy MHC-II-positive tumors. Based on our results, we believe that some of the keys to the success of an immunotherapy targeting MHC-II tumors are (a) proper activation of tumor-specific CD4+ T-cells and (b) identification of potential neo-antigens against which an efficient CD4+ T cell-based response can be induced.

Several groups have reported expression of MHC class II on human PDAC,^38,39,49^ although its functional role remains unclear. In other malignancies, for instance, melanoma, the engagement of MHC-II on MHC-II-positive melanoma cells was shown to protect the tumor cells from Fas-induced apoptosis,^37^ indicating that MHC-II is involved in the escape of melanoma cells from the immune system. Furthermore, it was shown that interaction between melanoma cells and activated lymphocytes via MHC-II/LAG-3 resulted in suppression of T-cells. This mechanism might explain why MHC-II expression on melanoma is associated with a poor prognosis. In contrast, our results demonstrate that MHC-II/LAG-3 interaction neither rescued PDAC cells from Fas-mediated apoptosis nor impaired the induction of activated CD4+ and CD8+ T-cells. In fact, inhibition of the MHC-II/LAG-3 axis impaired the effector function of T-cells. The fact that LAG-3 contributes to the effector functions of T-cells is contradictory to its described role as a checkpoint molecule to be targeted in certain cancers.^31–36^ In view of our findings, it is reasonable to suppose that LAG-3 plays different roles depending on the activation status of the T-cells, enhancing effector functions on activated T-cells, while promoting suppressive features on Treg or exhausted T-cells.

In tumors that do not express MHC-II, the participation of CD4+ T-cells in the local immune response relies on resident professional APCs that take up tumor cells and present their processed antigens on MHC-II to infiltrating CD4+ T helper cells. The CD4+ T-cells produce IL-2, IFN-γ and TNF-α to create an inflammatory micro-environment, favoring the activity of the infiltrating cytotoxic CD8+ T-cells (CTLs), thereby contributing to the elimination of the tumor.^50^ This indirect mechanism of action must be behind the success of adoptive transfer of *ex vivo* expanded tumor-specific CD4+ T-cells in promoting clinical remission of melanoma ^51^ and cholangiocarcinoma.^52^ A tumor expressing functional MHC-II molecules could potentially amplify the ongoing immune response, thus contributing to a more effective clearance of the tumor. Our data suggest that PDAC cells display functional MHC-II molecules loaded with tumor-derived peptides as PDAC cell lines induced to express MHC class II were killed by activated CD4+ T-cells.

A major issue in the host defense with consequences for immunotherapies is the tumor-immune-evasion,^53^ which may be a consequence of the downregulation of recognition structures on the cancer cells, such as MHC molecules.^38^ In this context, PDAC ^39^ is well known to downregulate the expression of MHC class I molecules as well as proteins of the MHC-I processing pathway, probably to escape a cytotoxic response mediated by CD8+ T-cells. However, in most cases, downregulation of MHC-I can be reversed by treatment with IFN-γ.^39^ In a number of clinical trials, for instance, in malignant melanoma,^54,55^ colon carcinoma^56^ and bladder cancer,^57^ IFN-γ treatment resulted in tumoral upregulation of MHC class I and *de novo* MHC class II expression and increased frequency of tumor-specific CD4+ T-cells. As MHC class II can be induced on PDAC cells, an IFN-γ-based therapy could be a valid option in the cases where there is no or low spontaneous tumoral expression of MHC-II. Although most PDAC patients and cell lines exhibit tumoral MHC-II expression, some failed to do so even under IFN-γ stimulation. A recent report showed that the co-treatment of lung cancer cells with IFN-γ and MEK and/or histone deacetylase (HDAC) inhibitors induced MHC-II expression in IFN-γ-resistant cells.^47^ In agreement with that, we observed expression of MHC-II in our PDAC cell lines when treated with IFN-γ in the presence of the MEK inhibitor trametinib and the HDAC inhibitor TSA. As trametinib is already utilized in clinical settings, its inclusion in immunotherapeutic protocols for PDAC would be feasible.

According to the current understanding, MHC class II molecules present processed exogenous antigens, whereas MHC class I presents endogenous self-antigens. However, various alternative pathways for antigen presentation of endogenous antigens on MHC class II have been proposed.^58–60^ Our data showing low expression of CLIP on the surface of PDAC cells, despite low HLA-DM, coupled to the cytotoxic activity of CD4+ T-cells toward MHC-II-positive PDAC cells, strengthen the argument that the MHC class II molecules are loaded with tumor-derived antigens, therefore potentially targetable. In view of these results, we postulate that MHC-II molecules on PDAC cells can be harnessed for neo-antigen-based immunotherapy. Neo-antigens, as a result of somatic mutations of tumors, have become attractive targets for immunotherapy. A major challenge in the development of a neo-antigen-based immunotherapy, addressed in this work, is the selection of the best targets, *i.e*., tumor-specific neo-antigens capable of eliciting robust immune responses. Current sequencing techniques and bioinformatic analysis tools enable rapid identification of somatic mutations from individual tumors. However, identification of viable neo-epitopes from a large pool of candidate peptides remains a significant hurdle to efficiently harness each patient’s unique set of neo-antigens for personalized immunotherapy. As reported in the literature and confirmed herein, the large majority of neo-antigens are unique, emphasizing the need for efficient approaches for rapid identification of personal neo-epitopes. Although prediction methods for HLA-binding affinity can narrow down the number of candidate neo-epitopes, they lack the accuracy to predict stability of the peptide–HLA complexes and immunogenicity. Through cell-based assays, we showed that only 67% of the predicted epitopes can in fact bind to the HLA allele. Moreover, not all binders were capable of eliciting a CD4+ T-cell-based response, irrespective of their binding capacity. This is in disagreement with some previous works showing a correlation between affinity of the peptide for the HLA and immunogenicity.^61,62^ Intriguingly, not all immunogenic epitopes triggered a CD4+ T-cell response in all donors tested. These observations indicate that immunogenicity of a peptide is mainly determined by the repertoire of T-cells with TCRs capable of recognizing that peptide – affinity being a secondary requirement. If any given peptide can bind to the HLA and remain exposed on the cell surface, it will induce a response so long as there are CD4+ T-cell clones endowed with the TCR for the MHC-II-peptide complex. Thus, while algorithms for the identification of binding peptides are improving, determining the immunogenicity of those peptides without the use of cell-based assays remains a challenge for the bioinformaticians.

Another major challenge for the selection of the best targets for a CD4+ T-cell-based immunotherapy is to identify which of the immunogenic neo-epitopes are generated by the MHC class II processing machinery and displayed by the tumor cells for T-cell recognition. Using exome and transcriptome data from Capan-1 cells and prediction algorithms, we selected 12 HLA-DP4-binding neo-antigen candidates, of which three were immunogenic to all donors tested. CD4+ T-cells induced against the three immunogenic epitopes were able to recognize MHC-II-positive Capan-1, indicating the presentation of those epitopes on the tumor cell surface. To our knowledge, this is the first demonstration of an approach that combines bioinformatics and cell-based tools to successfully identify immunogenic neo-antigens presented by human pancreatic cancer cells on MHC class II molecules.

It is important to note that our conclusions are based on *in vitro* experiments, which do not take into account the hostile tumor microenvironment of PDAC. Indeed, we believe that a neo-antigen-based immunotherapy alone would not be sufficient to impact tumor growth in PDAC patients. In a clinical setting, this strategy must be combined with TME-targeting strategies, which could be through systemic delivery of checkpoint inhibitors and/or oncolytic viruses to create a TME favorable to the action of the neo-antigen-specific T-cells mobilized by the vaccine.

One limitation of our study is that in all patients and cell lines analyzed, we could not identify shared epitopes, even when we considered only epitopes restricted by the common MHC-II allele HLA-DP4. As a consequence, CD4+ T-cell-based immunotherapy targeting MHC-II neo-antigens must be tailored to each individual patient as a personalized therapy.

In conclusion, this work shows that it is possible to generate cytotoxic CD4+ T-cells against PDAC cells expressing MHC-II molecules and the MHC-II/CD4+ T-cells axis should be a key focus of future immunotherapeutic strategies for PDAC. Moreover, we are confident that the platform for identification and screening of cancer-derived neo-antigens developed here is applicable and can be used to create a list of immunogenic peptides to be used as targets for personalized cancer immunotherapy. Pre-treatment with IFN-γ combined with MEK/ERK inhibitors and/or histone deacetylase inhibitors to enhance tumoral MHC-II expression could further expand the application of the immunotherapeutic approach proposed herein.

## Material and methods

### Imunohistochemistry

Pancreatic cancer tissues from 63 patients collected at cancer resection or biopsy at Barts Health NHS Trust (City and East London Research Ethics Committee 07/0705/87) as described previously^63,64^ were used to construct tissue microarrays (TMAs). The clinical characteristics of the human pancreatic cancer tissue microarray cohort are described elsewhere.^63,64^ Regions of tumor, stroma and normal pancreas were marked on H&E-stained slides of the donor tissue blocks, and three 1-mm cores of each region were sampled per patient using the Tissue Arrayer Minicore 3 (Alphelys, Plaisir, France). For immunohistochemical staining of the paraffin-embedded TMAs, 4 μm sections were first deparaffinized and re-hydrated. Antigen retrieval was done by heat-induced epitope retrieval in a pressure cooker using 10 mmol/L Citrate buffer at pH6.0 for 10 minutes. Peroxidase activity was blocked with 0.45% H_2_O_2_ in methanol, followed by protein blocking with serum. Expression of HLA-DM and MHC-II was assessed with anti-HLA-DM (Clone EPR7982; #ab131273; Abcam, United Kingdom) and anti-pan MHC-II (Clone CR3/43; #M0775; Dako). Primary antibodies were incubated for 1 h at room temperature and used at the following dilutions: anti-HLA-DM 1:500 and anti-MHC-II 1:2000. Biotinylated secondary antibody was added, and samples were incubated for 30 minutes at room temperature. Detection was done with peroxidase-labeled streptavidin (#P0397; Dako) and 3,3-diaminobenzidine (#D5637; Sigma-Aldrich) with 15 minutes incubation time. Tissues were counterstained with hematoxylin and the slides mounted for analysis. The number of tumor cells expressing the markers as well as the intensity of expression was assessed in at least three fields, at a magnification of 100x, under light microscopy. Thus, 0–5% positive cells (score 0), 5–25% positive tumor cells (score, 1), 25–50% positive tumor cells (score, 2), 50–75% positive tumor cells (score, 3) and more than 75% positive tumor cells (score, 4). The level of expression of the markers was scored according to the staining intensity of the positive cells: score 0, if no staining was detected; score 1, background staining; score 2, if the intensity was mild; score 3 if the intensity was moderate; and score 4 if the intensity was high. For the final score, the score of the percentage of positive cells was summed to the intensity of staining, ranging from 0 to 8. Macrophages and stromal cells in PDAC sections were used as positive and negative controls for both HLA-DM and MHC-II expressions, respectively. All analysis and scoring of the PDAC tissue samples were performed by an experienced member of our laboratory.

### Cell lines

Human primary PDAC cell lines PaTu8988T, ASPC-1, Capan-1, CFPAC-1, FA6, IMIMPC-2, MDA-Panc-3, MiaPaca-2, Panc-1, PT45, SUIT-2 were maintained in DMEM medium with 1% penicillin/streptomycin and 10% fetal bovine serum. PDAC circulating tumor cells CTC-76, CTC-102 and CTC-139, and lymphoblastoid T2 and T2-DP4 cells were cultured in RPMI-1640 medium with 1% penicillin/streptomycin and 10% fetal bovine serum. The primary cell lines were authenticated through short tandem repeat (STR) profiling. The circulating tumor cell lines were established in-house from late stage and chemo naive PDAC patients and were also authenticated through STR (Public Health England). To prevent phenotypic drift, after 50 passages for each cell line, a new vial was thawed. All cell lines were routinely tested for mycoplasma contamination and only used when mycoplasma-free.

### HLA-typing

The HLA-DP alleles of the PDAC cell line Capan-1 and all PBMC donors used for peptide-stimulation assays were determined by VH Bio Ltd (Gateshead, United Kingdom) using HLA typing with sequence-specific oligonucleotide primed PCR (PCR-SSO).

### IFN-γ stimulation

All PDAC cell lines were stimulated with 50 ng/mL of the human recombinant IFN-γ (#570206; BioLegend) in T75 culture flasks for 48 h. As negative control, we co-incubated the cell lines in the medium alone. Where inhibitors were used, trametinib (#S2673; Selleckchem) or trichostatin A (TSA) (#T8552-1 MG; Sigma-Aldrich) at the indicated concentrations were added into the cultures with or without IFN-γ. After incubation, the cells were removed from the flasks by treatment with trypsin, cell suspensions were prepared and cells were stained with purified mouse IgG antibodies to CLIP (clone CerCLIP.1; #ab22606; Abcam, Cambridge, UK), and HLA-DR, DP, DQ (clone Tü39; #555556; BD) or isotype control (mouse IgG2a; #554645; BD) followed by Alexa Fluor 488-conjugated anti-mouse IgG, and APC-conjugated anti-PD-L1 (clone 29E.2A3; #329708; BioLegend) and processed for flow cytometry analysis.

### Mitomycin c treatment of PDAC cell lines

PDAC cells were treated with mitomycin c to prevent their proliferation prior to co-culture with isolated T cells in T cell proliferation assays. Briefly, PDAC cell lines were incubated in a serum-free DMEM medium with 50 μg/mL of mitomycin c (ROCHE; #10107409001) for 1 h at 37°C. Following incubation, the cells were washed 4 times with a DMEM medium with 10% FBS and used in co-culture experiments with T-cells.

### Cell death assay

Cell death was studied in PDAC cells by incubation with 1 μg/mL anti-Fas antibody (clone EOS9.1; #305706; BioLegend) for 48 h. To see whether engagement of MHC class II on PDAC cells could protect them from cell death, PDAC cells were incubated with recombinant human soluble LAG-3 (sLAG-3) (IMP321; #2319-L3-050; R&D Systems) and/or anti-HLA-DR antibody (clone L243; #307648; BioLegend) at 10 μg/mL for 1 h. Then, anti-Fas antibody (1 μg/mL) was added for the following 48 h. Cell death was evaluated by staining the cells with Calcein-AM and ethidium homodimer 1 (EthD-1) (#L3224; Invitrogen, Carlsbad, CA, USA). Calcein-AM is a cell-permeable non-fluorescent probe that is metabolized into the fluorescent calcein in the cytosol of live cells. EthD-1 is a fluorescent probe that binds nucleic acid, but it cannot enter cells with intact cell membranes. PDAC cells treated with anti-Fas and/or sLAG-3/anti-HLA-DR were harvested and labeled with calcein-AM and EthD-1 for 20 min and analyzed by flow cytometry. The cells positive for calcein and negative for EthD-1 were considered alive and those positive for EthD-1 and/or negative for calcein, dead.

### Generation of DCs

Human DCs were differentiated as described elsewhere.^65^ Briefly, magnetic sorted CD14+ cells (Sorted with CD14 Microbeads Human, MACS Miltenyi; #130-050-201) from PBMCs of healthy donors were cultured in 75 cm^2^ cell culture flasks in RPMI 1640 GlutaMax culture medium (Invitrogen, Carlsbad, CA, USA) with 10% heat-inactivated fetal bovine serum (Invitrogen). For DCs differentiation, on day 0 and 4 the cultures were supplemented with 50 ng/mL recombinant human GM-CSF (#572904; BioLegend) and 50 ng/mL IL-4 (#574006; BioLegend). The cultures were maintained at 37°C in humidified atmosphere with 5% CO_2_. For DCs maturation, on day 5 of culture, LPS (#L2630-10 MG; Sigma-Aldrich, Germany) at 100 ng/mL was added to the iDCs and the culture continued for another 48 h. The cells were harvested and used for phenotyping through staining for the surface markers CLIP (clone CerCLIP.1; #ab22606; Abcam, Cambridge, UK), MHC-II (clone Tü39; #555556; BD), CD80 (#557226; BD), CD83 (#556855; BD) and CD86 (#555660; BD) or in co-cultures with T-cells.

### Activation of T-cells

For T cell activation, PBMCs were cultured for 5 days with anti-human CD3/CD28-coated beads (Dynabeads™ Human T-Activator CD3/CD28 for T Cell Expansion and Activation; #11132D; Invitrogen) and 5 ng/mL human recombinant IL-12 (#573002; BioLegend). Human recombinant IL-2 (#589106; BioLegend) at 30 U/mL and IL-15 (#570304; BioLegend) at 5 ng/mL was added on day 2 and the culture continued. On day 5, beads were removed and the cells were maintained in culture for another 48 h with IL-2 (10 U/mL) and IL-15 (5 ng/mL). Cells were stained with fluorochrome-labeled antibodies to CD4 (clone OKT4; #317432; BioLegend), CD8 (clone HIT8a; #300918; BioLegend) and LAG-3 (clone 11C3C65; #369326; BioLegend) and analyzed by flow cytometry or used in functional assays.

### T-cell proliferation assay

CD4+ and CD8+ T-cells were magnetically sorted (CD4 Microbeads Human; #130-045-101 and CD8 Microbeads Human; #130-045-201; Miltenyi Biotech), stained with 5 µM CFSE (#V12883; Invitrogen, Carlsbad, CA, USA) and incubated with mitomycin c-treated PDAC cell lines in U-bottom 96 well plates for 4 days. Allogeneic mature DCs were used as positive controls. Where indicated, purified endotoxin-free anti-MHC class I (clone W6/32; #311428; BioLegend), anti-MHC class II (clone TÜ39; #555556; BD Bioscience, Chicago, IL, USA), anti-LAG-3 (clone 17B4; Novus Biologicals; #NBP1-97657), anti-PD-L1 (clone 29E.2A3; #329716; BioLegend) or a combination of anti-LAG-3 and anti-PD-L1 antibodies were added at 10 μg/mL. The results are expressed as proliferation index, determined by dividing the mean fluorescence intensity (MFI) of CFSE in T-cells alone by the MFI of the T-cells in the co-cultures. Supernatants were collected for the assessment of IFN-γ and granzyme B by ELISA. Cells were stained with fluorochrome-labeled anti-CD4 (#317422; BioLegend), anti-CD8 (#300918; BioLegend) and anti-perforin (#353305; BioLegend) and processed for flow cytometry.

### Cytotoxicity assays

To measure cytotoxicity in the allogeneic assays, purified activated CD4+ or CD8+ T-cells from HLA-A*02:01-negative donors were incubated with HLA-A*02:01-positive PDAC cell lines at a ratio of 2:1. Where indicated, soluble LAG-3 (#2319-L3-050; R&D Systems), anti-LAG-3 (clone 17B4; Novus Biologicals; #NBP1-97657), anti-PD-L1 (clone 29E.2A3; #329716; BioLegend) or a combination of anti-LAG-3 and anti-PD-L1 antibodies or soluble LAG-3 and anti-PD-L1 were added at 10 μg/mL. After overnight incubation, the cells were stained with BV421-conjugated anti-HLA-A2 (#740082; BD), Calcein-AM and Ethidium Homodimer 1 (#L3224; Invitrogen) to determine cell death. Target PDAC cells were gated on HLA-A2 positive cells. The cytotoxicity of peptide-stimulated CD4+ T-cells on Capan-1 cells was determined through measurement of lactate dehydrogenase (LDH) released into the culture medium with the CytoTox 96® Non-Radioactive Cytotoxicity Assay Kit (#G1780; Promega). The assay was carried out according to the manufacturer’s instructions. Results are expressed as relative cytotoxicity, that is, cell death of Capan-1 cells by peptide-stimulated CD4+ T-cells in relation to unstimulated CD4+ T-cells.

## Elisa

The cytokine IFN-γ and the cytolytic molecules granulysin and granzyme B were detected in supernatants of T-cell/tumor cell co-cultures using commercial ELISA kits (Human IFN gamma ELISA Ready-SET-Go!®, #88-7316-76, Affymetrix eBioscience; Human Granulysin DuoSet ELISA, #DY3138; and Human Granzyme B DuoSet ELISA, #DY2906-05, R&D Systems) in 96-well microtiter plates (Nunc Maxisorp, Rochester, NY, USA) following the manufacturer’s instructions.

### Prediction of potential neo-epitopes from PDAC patients

Neo-epitopes for 127 PDAC patients in TCGA-PAAD were identified from whole-exome sequencing data with pVactools.^66^ Germline mutations were detected from matched normal tissue sequencing using GATK haplotype caller and CNN Score Variants. Varscan2 detected somatic variants were downloaded, annotated with Ensembl Variant Effect Predictor ^67^ and gene expression data was added. A phased VCF file was generated by combining germline and somatic variant calls using GATK Combine Variants and GATK Read Backed Phasing. Patient HLA haplotypes were acquired with Seq2HLA ^68^ and used as input for pVactools’ pvacseq. Candidate neo-epitopes were filtered on median affinity and DNA variant allele frequency (min 0.25).

### Identification of neo-epitopes from PDAC patients for validation in cell-based and immunogenicity assays

For the identification of potential neo-epitopes from the somatic missense mutations detected from exome sequencing analysis of 100 PDAC patients,^46^ exome sequencing data were used to compile a list of expressed somatic missense mutations. Amino-acid substitutions corresponding to each of the coding missense mutations were translated into a 29-mer amino acid FASTA sequence, with 14 amino acids flanking the mutated amino acid on each side. These 29-mer amino-acid sequences were evaluated through the MHC class II peptide–binding algorithm NetMHCIIpan-3.0 ^69^ to identify high-affinity 15mer, neo-epitopes predicted to bind with high affinity to HLA-DP*04:01. Neo-epitopes were selected when mutated peptide fell within the 20% percentile rank and the corresponding wild type was above the 20%.

### Identification of neo-epitopes from capan-1

Whole-exome sequencing was performed according to GATK best practices.^70^ Briefly, an unmapped BAM file was generated from raw fastq sequencing output. Adapter sequences were removed, and the sequence reads were mapped to GRch38 with Burrows-Wheeler Aligner.^71^ Duplicates were marked with Mark Duplicates, and base quality scores were recalibrated using known sites from 1000 G,^72^ dbsnp^73^ and hapmap^74^ as references. Candidate variants were identified with GATK Mutect2, filtered with Filter Mutect Calls and annotated with Ensembl Variant Effect Predictor. RNA sequencing was performed with STAR,^75^ following quality control with fastqc reads were aligned with STAR and expression was calculated with RSEM.^76^ Neo-antigen discovery was performed according to pVactools’ protocol. Gene expression data was added to variants and 15-mer peptides with affinity to HLA-DPA1*01, DPB1*04:01 were generated with pvactools (version 2.alpha.7). Affinity was determined as the median score of antigens in MHCnuggetsII, NNalign, netMHCIIpan and SMMalign, candidates were filtered on expression (FPKM <1) and variant allele frequency and coverage filters were disabled. Sequencing data of Capan-1 was deposited in a repository (https://www.ncbi.nlm.nih.gov/bioproject), accession number PRJNA778344.

### Peptides

All peptides were purchased from ProImmune Ltd (Oxford, United Kingdom) or GL Biochem Ltd. (Shanghai, China). Peptides were custom-synthesized with a purity >95%. Lyophilized peptides were dissolved in DMSO (Pierce, Rockford, Illinois, USA) at 20 mg/mL and stored at −80°C until use. Peptides were never used more than once after thawing.

### Cell-based competitive MHC-II binding assay

Binding of HLA-DP4 neo-epitopes was assessed in a cell-based competitive binding assay with lymphoblastoid T2 cell line transfected with HLA-DP4 (T2-DP4; kindly provided by Professor Naoto Hirano from the University of Toronto). T2-DP4 cells were grown and maintained in culture in RPMI medium with 10% FCS at 37°C with 5% CO_2_. For the cell-based competitive binding assay, T2-DP4 cells were incubated in RPMI medium with 10% FCS overnight at 37°C, with peptide concentrations ranging from 80 µM (in 8 serial dilution steps) in the presence of 500 nM of FITC-labeled reference peptide Oxy_271-287_ (EKKYFAATQFEPLAARL).^77^ Positive and negative controls were TT_947-967_ (FNNFTVSFWLRVPKVSASHLE) and MUC-1_140-148_ (SAPDTRPAP), respectively. The relative binding of the unlabeled competitor peptides was expressed as inhibitory concentration (IC50), *i.e*., the concentration of competitor peptide required to inhibit 50% of binding of the FITC-labeled reference peptide. All measurements were performed with a FACSCalibur flow cytometer (BD Bioscience, Heidelberg, Germany) or BD LSRII (Becton Dickinson). Data analysis was done with WinMDi 2.9 (Purdue University, USA), and IC50 calculation with GraphPad Prism version 5 for Windows (GraphPad Software, La Jolla, CA).

### Immunogenicity T-cell assay and generation of neo-antigen-specific CD4+ T-cells

Immunogenicity of candidate HLA-DP4 neo-epitopes was performed using HLA-matched healthy donors’ autologous PBMCs. Briefly, mature DCs were pulsed with individual neo-epitopes at 10 μg/mL and co-cultured with autologous magnetic-sorted CD4+ T-cells (CD4 Microbeads Human, MACS Miltenyi; #130-045-101) in RPMI with 5% AB human serum (#H4522-100ML; Sigma-Aldrich), 10 units/mL penicillin–streptomycin, 2 mmol/L L-glutamine, 1% nonessential amino acid, IL-6 (#570802; BioLegend) (1000 U/mL) and IL-12 (#573002; BioLegend) (5 ng/mL). IL-2 (#589106; BioLegend) (30 U/mL) and IL-7 (#581902; BioLegend) (5 ng/mL) was added every 2 days. CD4+ T-cells underwent another two rounds of stimulation with peptide-pulsed mature DCs on day 7 and 14. Seven days after the third stimulation, CD4+ T-cells were challenged by overnight incubation with peptide-loaded T2-DP4 cells. As a control, CD4+ T-cells were incubated with T2-DP4 without peptide. Reactivity of the T cells was determined by intracellular IFN-γ (#502515; BioLegend) staining measured by flow cytometry. When Capan-1-derived neo-epitopes were tested, the peptide-stimulated CD4+ T-cells were challenged by overnight incubation with Capan-1 (MHC-II-negative), IFN-γ-treated Capan-1 cells (MHC-II-positive). Supernatants were collected and assessed for LDH (cytotoxicity), IFN-γ, granzyme B and granulysin. Where indicated, purified endotoxin-free anti-MHC class II (clone TÜ39; #555556; BD Bioscience, Chicago, IL, USA) antibodies were added to the co-cultures at 10 μg/mL.

### Flow cytometry

For cell surface labeling, cell suspensions were incubated with antibodies for 30 min at room temperature. For intracellular staining, cells were fixed and permeabilized using a Leucoperm kit (Bio-Rad; #BUF09C) in accordance with the manufacturer’s guidelines. Prior to intracellular staining with anti-IFN-γ, cells were incubated overnight in the presence of 5 microgram per mL brefeldin A (Sigma-Aldrich; #B6542-5 MG). Cell viability was analyzed by Ethidium homodimer 1 and calcein-AM double-staining using LIVE/DEAD® Viability/Cytotoxicity Kit, for mammalian cells according to the manufacturer’s instructions (#L3224; Invitrogen). Flow cytometry was done using a FACSCalibur (Becton Dickinson, Heidelberg, Germany) or BD LSRII (Becton Dickinson) flow cytometer. The data were processed with the CellQuest software (Becton Dickinson, Heidelberg, Germany) and analyzed with the WinMDi 2.9 (Purdue University, USA).

### Statistical analysis

Statistical analysis was carried out using GraphPad Prism version 5. Two-tailed unpaired or paired t-tests between two groups and one-way analysis of variance (ANOVA) across multiple groups followed by Dunnett's as post test were used to determine significance. Data represent means ± SEM. P < 0.05 was considered significant (*P < 0.05; **P < 0.01; ***P < 0.001; ****P < 0.0001).

### Ethics statement

This study was reviewed and approved by the London-Westminster Research Ethics Committee (REC reference: 16/LO/1512) for access to healthy donor blood via the NHS Blood and Transplant Service. The collection of blood from PDAC patients for isolation of circulating tumor cells (CTC lines) was performed under the Barts Pancreas Tissue Bank Protocol, REC reference: 13/SC/0592. All human materials used had written informed consent.

## Supplementary Material

Supplemental Material

## Data and materials availability

All data needed to evaluate the conclusions in the paper are present in this article or the supplementary materials. All materials may be made available to the scientific community upon request.
