# Supplementary material for: MHC class II molecules on pancreatic cancer cells indicate a potential for neo-antigen-based immunotherapy
Source: Oncoimmunology. 2022 May 27;11(1):2080329. doi: 10.1080/2162402X.2022.2080329 (PMC9154752; doi:10.1080/2162402X.2022.2080329)
Supplement: Supplemental Material [file KONI_A_2080329_SM1578.docx]

**Supplementary Figures and Tables**

Supplementary Material


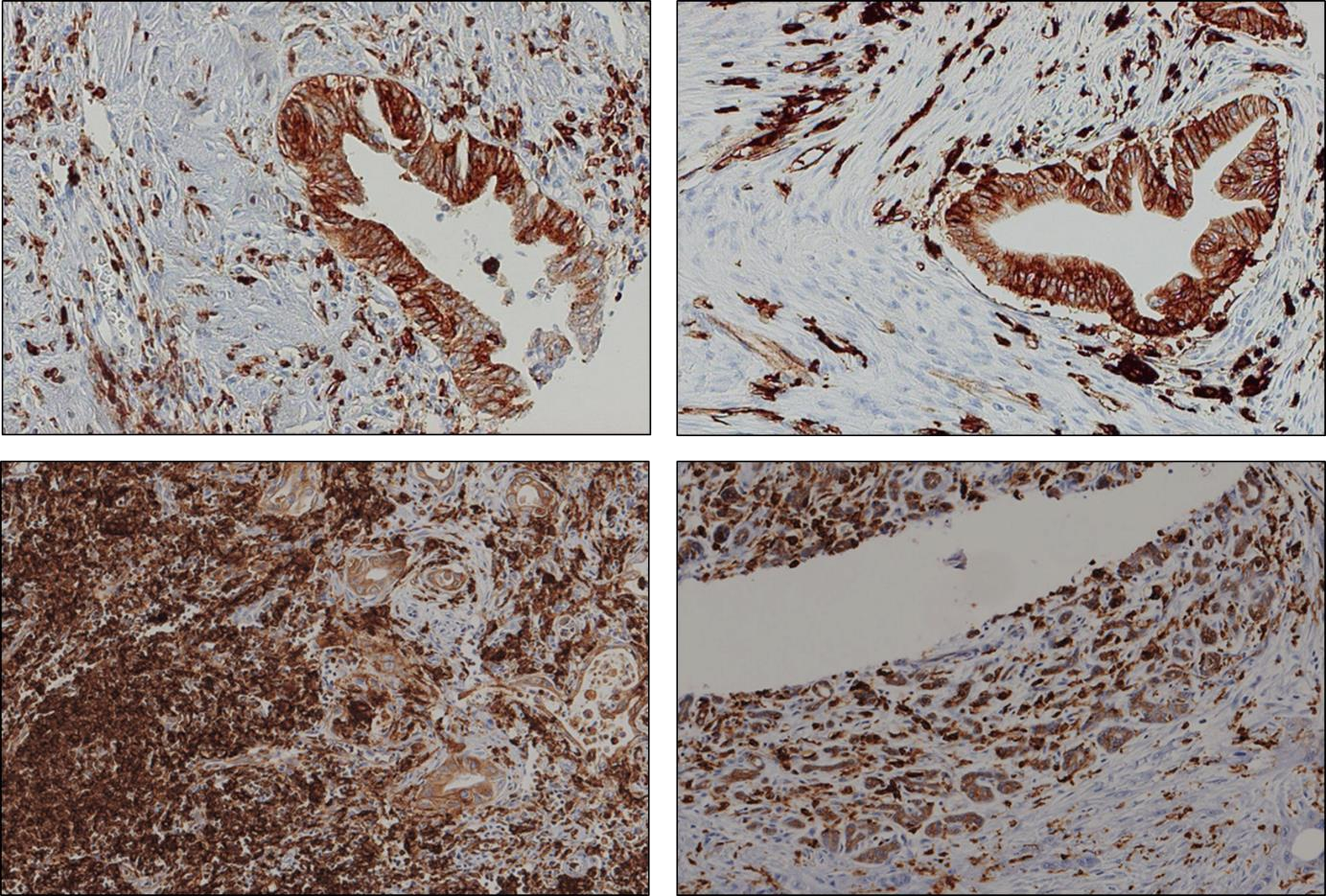


**Fig. S1. Immune infiltrates in PDAC samples with tumoral MHC-II expression.** Expression of MHC-II in paraffin-embedded sections of human PDAC was determined by immunohistochemistry using anti-pan MHC-II antibody (clone CR3/43) as described in Materials and Methods. Note expression of MHC-II on epithelial PDAC cells as well as immune infiltrating cells. Representative sections of samples from four different PDAC patients are shown. Magnification of images: 10x plus electronic zoom.


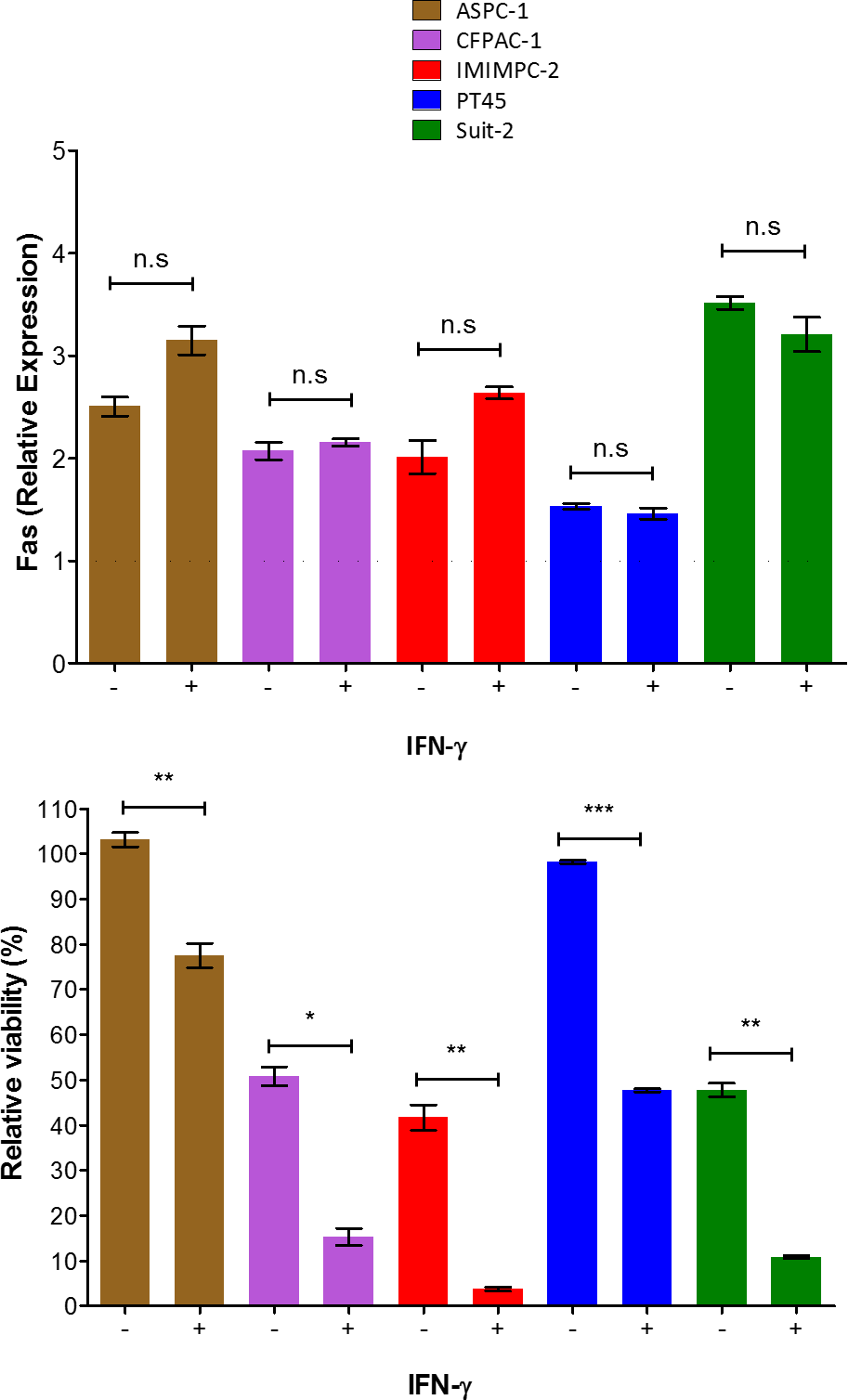


**Fig. S2. Expression of Fas on PDAC cell lines and their susceptibility to Fas-induced cell death.** PDAC cell lines were incubated for 48 hours in the presence or absence of IFN-γ and Fas expression was determined by flow cytometry (upper graph). Untreated or IFN-γ-treated cells were incubated with anti-Fas antibody for 48 hours and cell viability determined by staining with EthD-1 and calcein-AM (lower graph). The graphs show the means of 3 independent experiments and S.E.M. Data were analyzed by two-tailed paired t test. *P< 0.05; **p< 0.01; ***p< 0.001; n.s: non- significant.


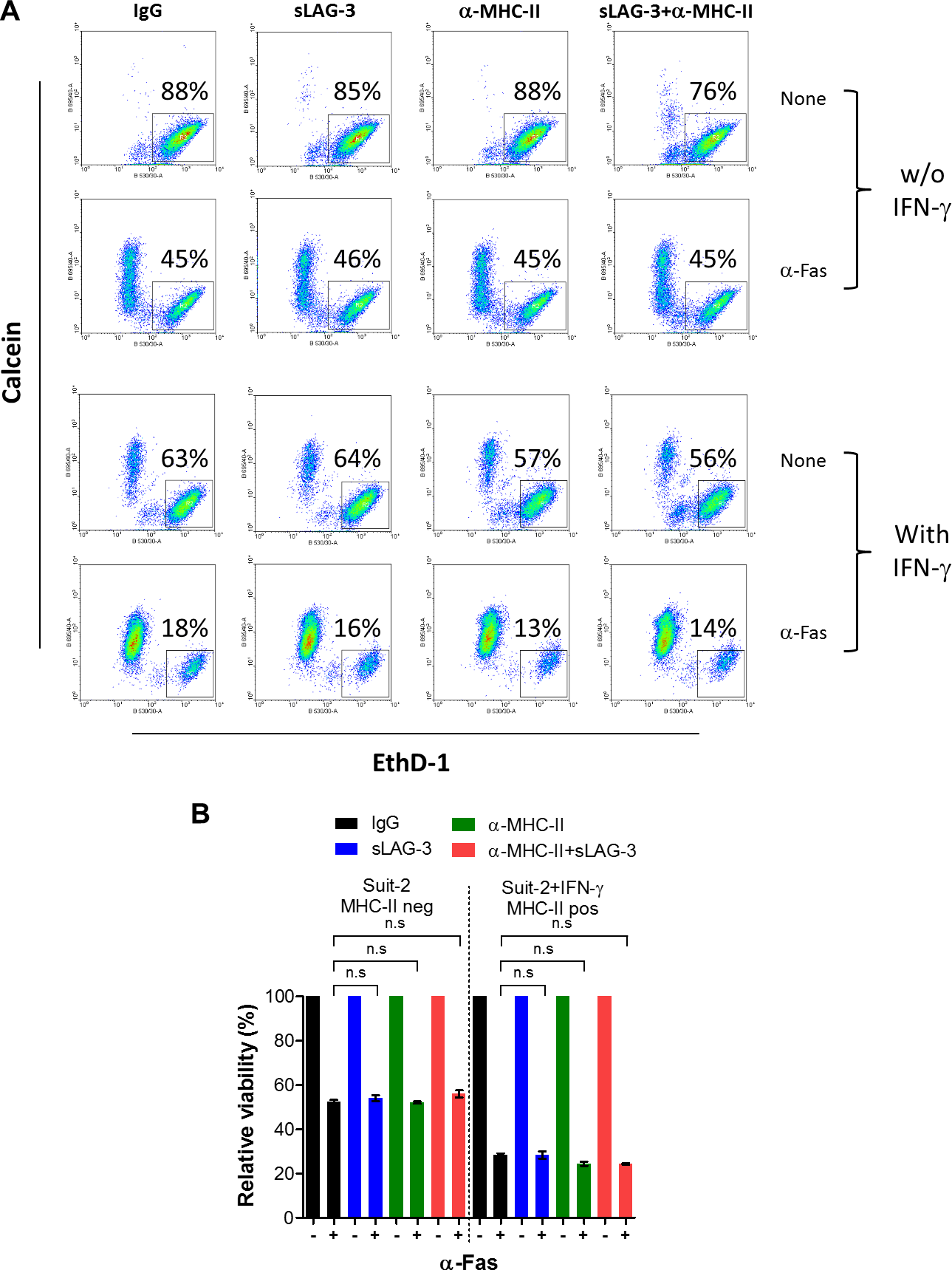


**Fig. S3. Engagement of MHC-II by soluble LAG-3 or agonistic antibody to MHC-II does not protect MHC-II-positive Suit-2 PDAC cells from Fas-induced cell death.** Fas-sensitive MHC-II- negative and -positive Suit-2 cells were cultured in the presence of irrelevant IgG isotype control, sLAG-3, agonistic anti-MHC-II antibody (clone L243) or sLAG-3 plus anti-MHC-II for 1 h, then treated or not with anti-Fas antobody (clone EOS9.1) for 48 h. Cell death was determined by staining with EthD-1 and calcein-AM followed by flow cytometry analysis. Dot plots of one representative experiment is shown in (**A**). Results are presented as mean percentages and S.E.M of viable cells from three independent experiments (**B**). Data were analyzed by two-tailed paired t test. n.s: non- significant.


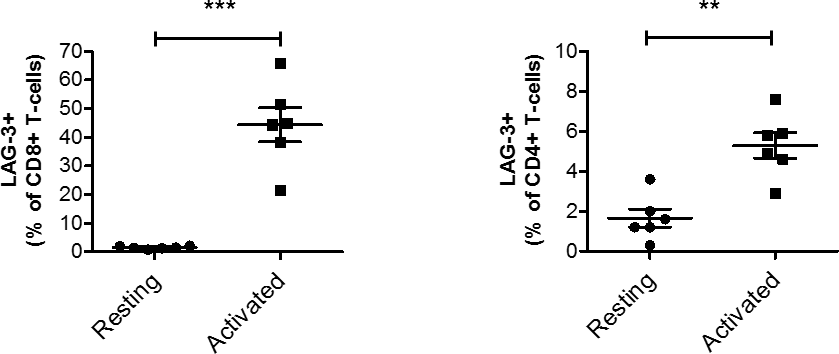


**Fig. S4. Activated T lymphocytes express LAG-3.** For T-cell activation, PBMCs from healthy donors were incubated with anti-CD3/CD28-coated beads plus IL-12. On day 2, cells were supplemented with IL-2 and IL-15 and the culture continued for another 3 days. Following 5-day culture period, beads were removed and cells were allowed to rest for 48 hours in the presence of IL- 2 and IL-15. Resting cells were maintained in culture medium without beads and cytokines for the whole culture period. Cells were then stained with fluorochrome-conjugated antibodies to CD3, CD4, CD8 and LAG-3 and analyzed by flow cytometry. Results are presented as mean percentages and

S.E.M of LAG-3 positive cells among the CD8+ (right) or CD4+ (left) T lymphocytes. Statistical significance between resting and activated T-cells was determined by two-tailed paired t test. **p< 0.01; ***p< 0.001.


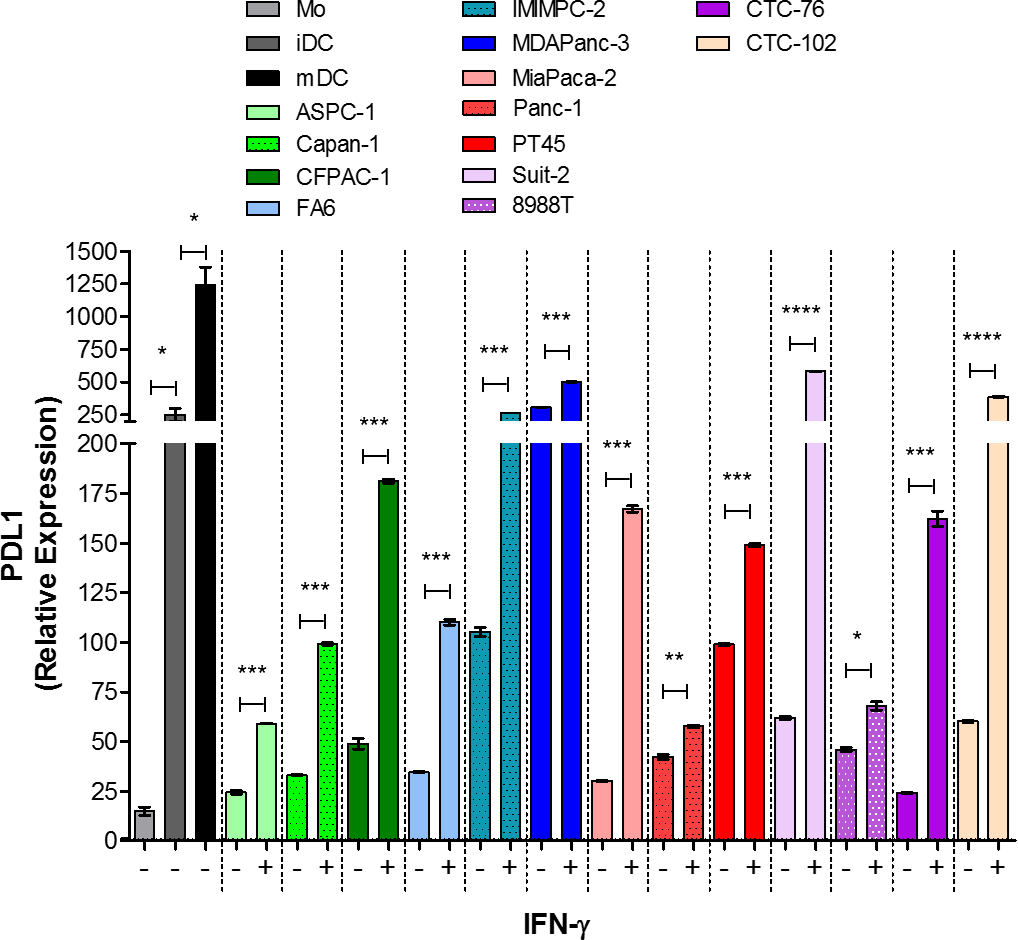


**Fig. S5. Expression of PD-L1 on PDAC cell lines.** PDAC cell lines were incubated for 48 hours in the presence or absence of IFN-
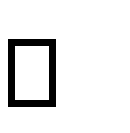
and cell-surface PD-L1 expression was determined by flow cytometry. Monocytes (Mo), immature and mature dendritic cells (DC) were used as controls. The graphs show the means of 3 independent experiments and standard error of the mean. Data were analyzed by two-tailed paired t test. *P< 0.05; **p< 0.01; ***p< 0.001; ****p<0.0001; n.s: nonsignificant.


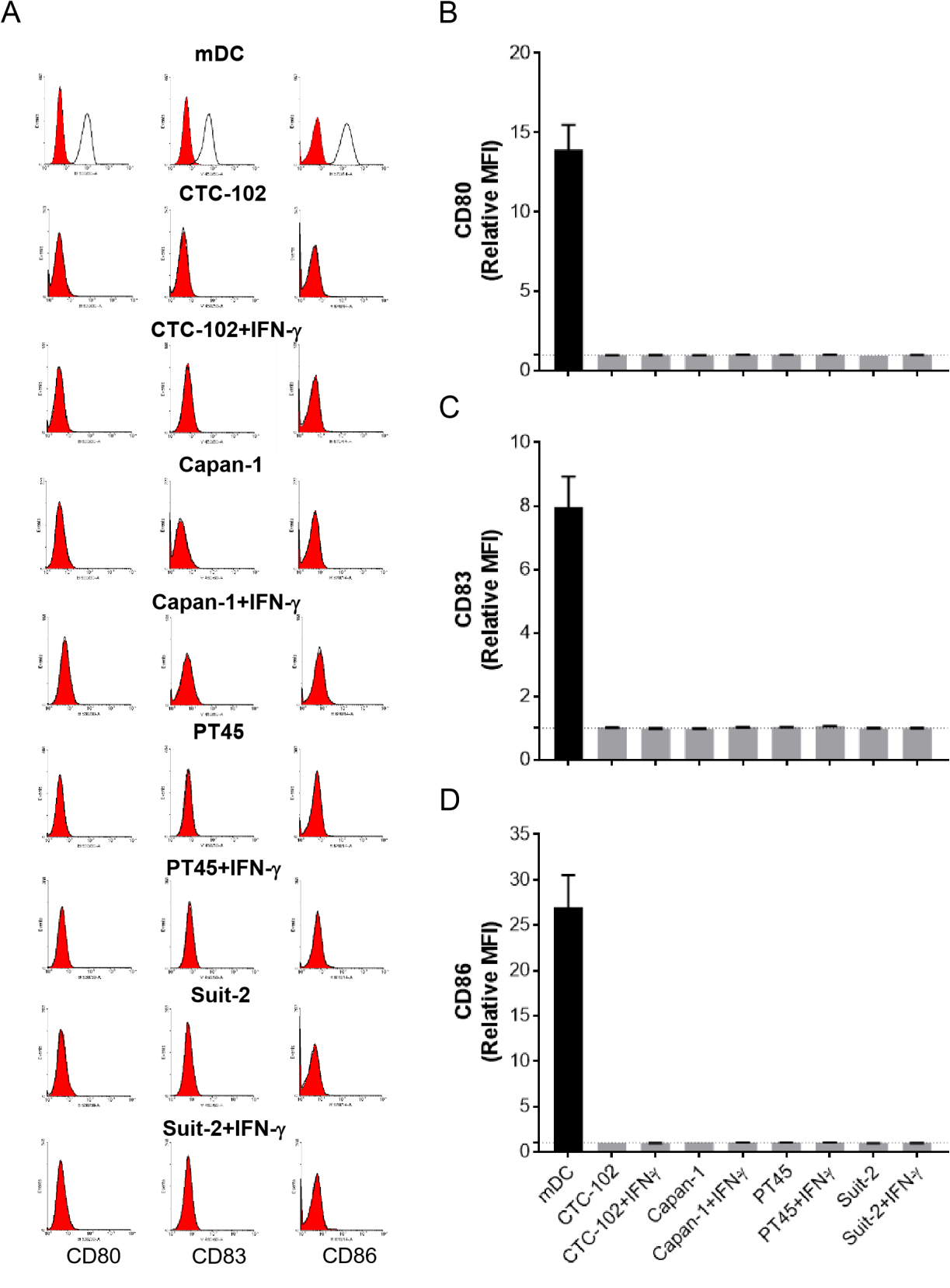


**Fig. S6. Expression of the co-stimulatory molecules CD80, CD83 and CD86 on mDC and PDAC cell lines.** The indicated PDAC cell lines were incubated for 48 hours in the presence or absence of IFN-γ and cell-surface CD80, CD83 and CD86 expression was determined by flow cytometry. mDC were used as positive control. Histograms in (A) is a representative experiment. Red-filled histogram:

unstained cells; Black line histogram: stained cells. The graphs show the means of 5 independent experiments and standard error of the mean for CDD80 (B), CD83 (C) and CD86 (D).


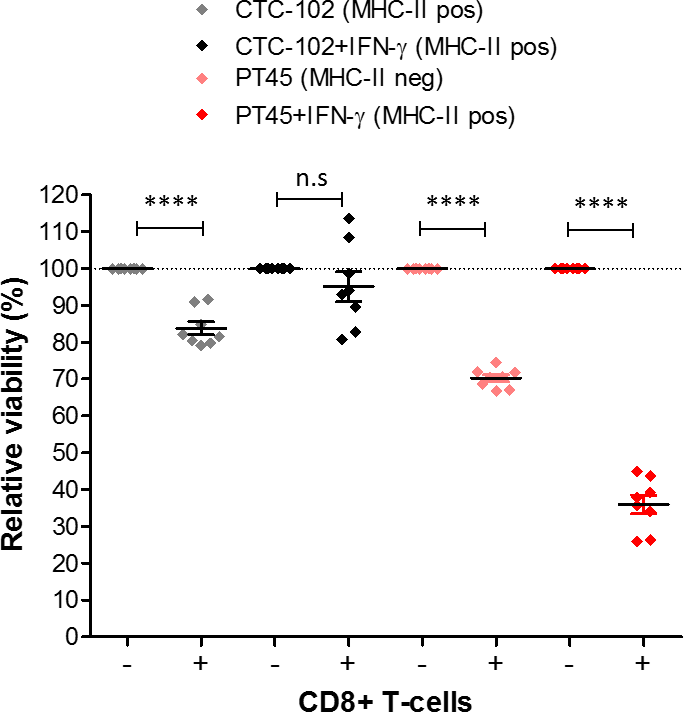


**Fig. S7. Cytotoxic activity of activated CD8+ T-cells.** Activated CD8+ T-cells from HLA- A2negative donors were incubated overnight with untreated or IFN-γ-treated HLA-A2-positive PDAC lines PT45 and CTC-102. Cell viability of PDAC cells was determined by staining cells with EthD-1, calcein-AM and BV421-conjugated anti-HLA-A2 antibody followed by flow cytometry analysis. PDAC cells were gated on the HLA-A2-positive population. Results are presented as mean percentages and S.E.M of viable cells from four donors performed in duplicate. Data were analyzed by two-tailed paired t test. ****p< 0.0001; n.s: non-significant.


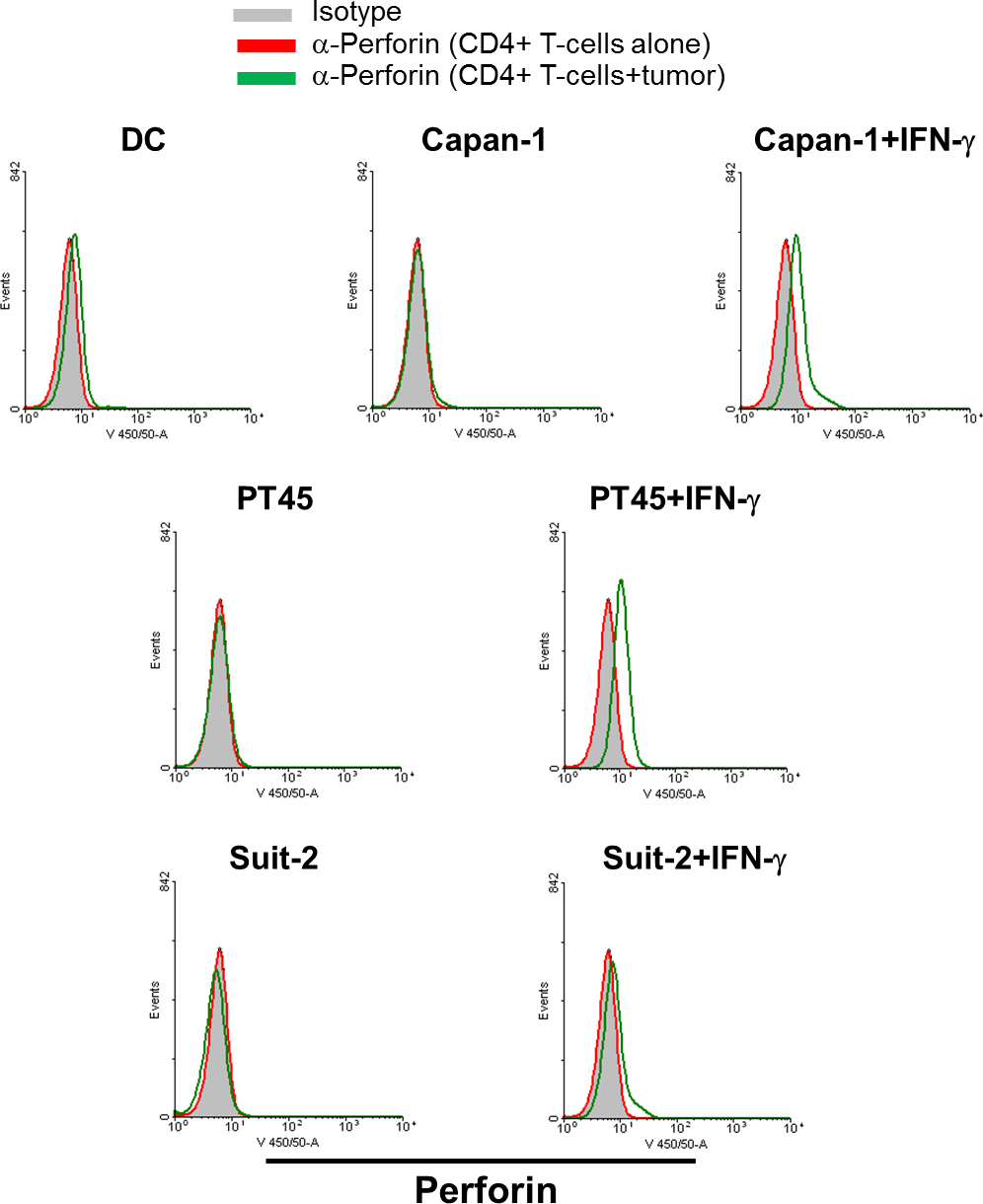


**Fig. S8. Cytotoxic activity of activated CD8+ T-cells**. Allogenic activated CD4+ T-cells from healthy donors were incubated for four days with PDAC cells (T-cell:tumor ratio 2:1). Intracellular perforin in CD4+ T-cells from these co-cultures was determined by flow cytometry. Allogenic mature DCs (T cell:DC ratio 10:1) were used as stimulator of CD4+ T-cells as positive control. Representative histograms from one out of 4 experiments are shown.


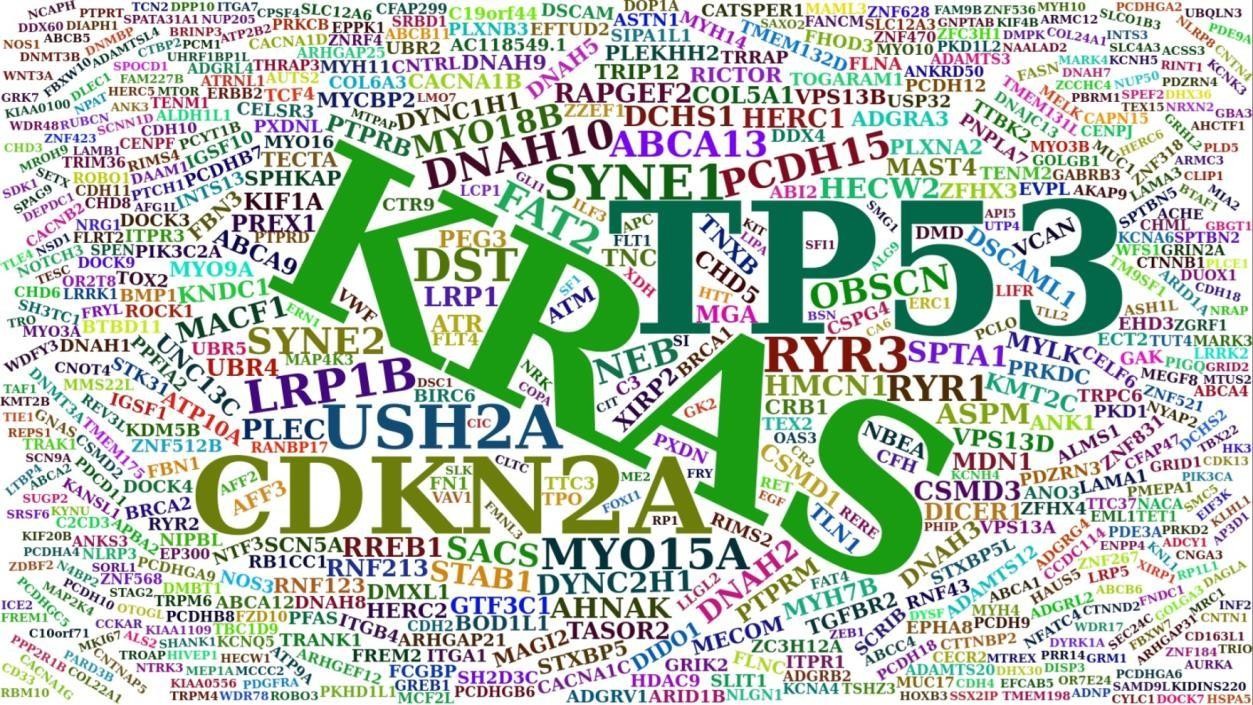


**Fig. S9. Wordcloud depicting the mutations found in PDAC patients_._**

**F IT C - O x y**

**2 7 1 - 2 8 7**

**( R e la t iv e M F I)**

#
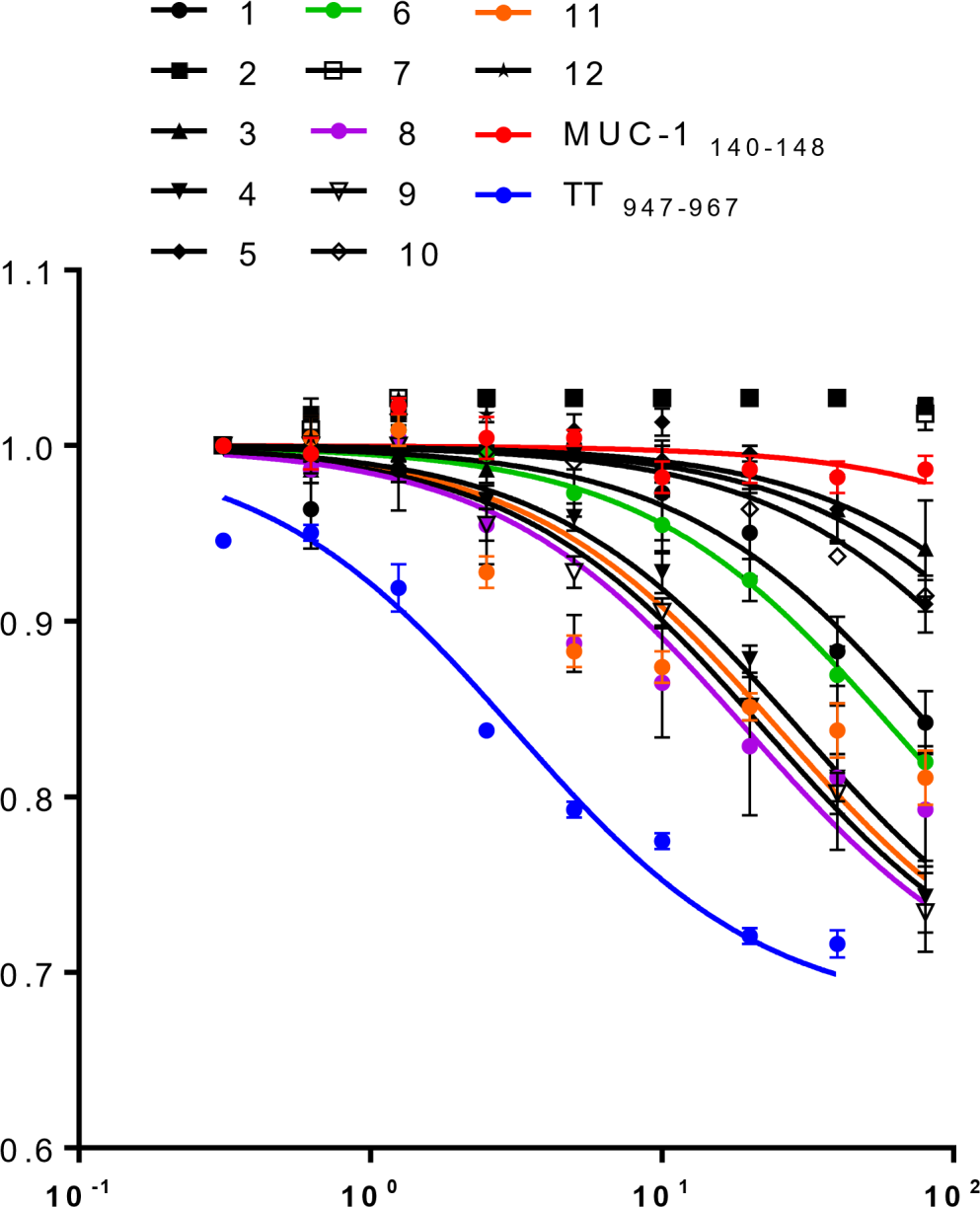
Competitor peptide [μM ]

**Fig. S10. Binding capacity of Capan-1 neo-antigen peptides.** Twelve peptides representing neo- antigens with good predicted binding affinity to HLA-DP4 molecules were examined in a competitive binding assay using T2 cells transfected with HLA-DP4 (T2-DP4). T2-DP4 cells were incubated with the test peptides in the presence of 0.5 μM FITC-conjugated reference peptide, Oxy _271-287_. n=3 per peptide. S.E.M is shown. MUC-1 _140-148_ and TT _947-967_ peptides were used as negative and positive control respectively; TT: Tetanus toxin.

**Table S1A.** Immunohistochemistry analysis of the expression of MHC-II on

PDAC cells and their differentiation status

| **Patient** | **Score for (%) positive**  **cells** | **Score for**  **intensity of staining** | **Final score** | **Tissue differentiation**  **status** |
| --- | --- | --- | --- | --- |
| 1 | 2 | 2.5 | 4.5 | Moderate |
| 2 | 1 | 1 | 2 | Moderate |
| 3 | 0 | 0 | 0 | Well |
| 4 | 2 | 3 | 5 | Moderate |
| 5 | 1 | 1 | 2 | Moderate |
| 6 | 1.7 | 2.3 | 4 | Well |
| 7 | 0.6 | 1.8 | 2.4 | Poor |
| 8 | 1 | 2 | 3 | Well |
| 9 | 2 | 3 | 5 | Moderate |
| 10 | 1.6 | 2.6 | 4.2 | Well |
| 11 | 1 | 2 | 3 | Well |
| 12 | 0 | 0 | 0 | Well |
| 13 | 3 | 3.5 | 6.5 | Moderate |
| 14 | 2.5 | 3.5 | 6 | Moderate |
| 15 | 2 | 2.5 | 4.5 | Moderate |
| 16 | 5 | 2.5 | 7.5 | Moderate |
| 17 | 2.5 | 2 | 4.5 | Moderate |
| 18 | 0 | 0 | 0 | Well |
| 19 | 0 | 0 | 0 | Well |
| 20 | 1 | 2.5 | 3.5 | Moderate |
| 21 | 3 | 4 | 7 | Well |
| 22 | 1.6 | 2 | 3.6 | Moderate |
| 23 | 1 | 1.3 | 2.3 | Well |
| 24 | 2.5 | 3 | 5.5 | Moderate |
| 25 | 1 | 1 | 2 | Well |
| 26 | 2.3 | 4 | 6.3 | Moderate |
| 27 | 2 | 2.5 | 4.5 | Moderate |
| 28 | 2.5 | 2.5 | 5 | Moderate |
| 29 | 2 | 2 | 4 | Poor |
| 30 | 3 | 3 | 6 | Well |
| 31 | 2.3 | 2 | 4.3 | Well |
| 32 | 1.5 | 2 | 3.5 | Well |
| 33 | 0 | 0 | 0 | Well |
| 34 | 0 | 0 | 0 | Well |
| 35 | 1 | 1 | 2 | Well |
| 36 | 0 | 0 | 0 | Moderate |
| 37 | 2 | 1.6 | 3.6 | Moderate |
| 38 | 0 | 0 | 0 | Moderate |
| 39 | 2.3 | 2 | 4.3 | Moderate |
| 40 | 2 | 2 | 4 | Well |
| 41 | 0 | 0 | 0 | Well |
| 42 | 1 | 1.5 | 2.5 | Moderate |
| 43 | 0 | 0 | 0 | Moderate |
| 44 | 2.5 | 3 | 5.5 | Moderate |
| 45 | 0 | 0 | 0 | Moderate |
| 46 | 0 | 0 | 0 | Well |
| 47 | 2 | 3 | 5 | Moderate |
| 48 | 0 | 2 | 2 | Well |
| 49 | 0 | 0 | 0 | Moderate |
| 50 | 0 | 0 | 0 | Moderate |
| 51 | 1.6 | 1 | 2.6 | Poor |
| 52 | 1.6 | 2 | 3.6 | Moderate |
| 53 | 1 | 2 | 3 | Moderate |
| 54 | 1.5 | 3 | 4.5 | Well |
| 55 | 3.4 | 3.8 | 7.2 | Well |
| 56 | 1.3 | 1.3 | 2.6 | Well |
| 57 | 2 | 2 | 4 | Well |

| 58 | 2 | 2 | 4 | Moderate |
| --- | --- | --- | --- | --- |
| 59 | 0 | 0 | 0 | Well |
| 60 | 1.5 | 1.5 | 3 | Well |
| 61 | 1 | 1 | 2 | Moderate |
| 62 | 1 | 1 | 2 | Well |
| 63 | 0 | 0 | 0 | Poor |

**Table S1B.** Immunohistochemistry analysis of the expression of HLA-DM by

PDAC cells

| **Patient** | **Score for (%) positive**  **cells** | **Score for intensity of**  **staining** | **Final score** |
| --- | --- | --- | --- |
| 1 | 1 | 0.5 | 1.5 |
| 2 | 0 | 0 | 0 |
| 3 | 0 | 0 | 0 |
| 4 | 0 | 0 | 0 |
| 5 | 0 | 0 | 0 |
| 6 | 0 | 0 | 0 |
| 7 | 0 | 0 | 0 |
| 8 | 0 | 0 | 0 |
| 9 | 0 | 0 | 0 |
| 10 | 1 | 0.5 | 1.5 |
| 11 | 0 | 0 | 0 |
| 12 | 0 | 0 | 0 |
| 13 | 0 | 0 | 0 |
| 14 | 0 | 0 | 0 |
| 15 | 0 | 0 | 0 |
| 16 | 0 | 0 | 0 |
| 17 | 0 | 0 | 0 |
| 18 | 0 | 0 | 0 |
| 19 | 0 | 0 | 0 |
| 20 | 0 | 0 | 0 |
| 21 | 2 | 0 | 2 |
| 22 | 0 | 0 | 0 |
| 23 | 0 | 0 | 0 |
| 24 | 0 | 0 | 0 |
| 25 | 0 | 0 | 0 |
| 26 | 0 | 0 | 0 |
| 27 | 2 | 1 | 3 |
| 28 | 1 | 0 | 1 |
| 29 | 1 | 0 | 1 |
| 30 | 0 | 0 | 0 |
| 31 | 0 | 0 | 0 |
| 32 | 0 | 0 | 0 |
| 33 | 0 | 0 | 0 |
| 34 | 0 | 0 | 0 |
| 35 | 1 | 0 | 1 |
| 36 | 0 | 0 | 0 |
| 37 | 1 | 0 | 1 |
| 38 | 0 | 0 | 0 |
| 39 | 1 | 1 | 2 |
| 40 | 0 | 0 | 0 |

| 41 | 0 | 0 | 0 |
| --- | --- | --- | --- |
| 42 | 0 | 0 | 0 |
| 43 | 0 | 0 | 0 |
| 44 | 1 | 1 | 2 |
| 45 | 0 | 0 | 0 |
| 46 | n.d | n.d | n.d |
| 47 | 1 | 0 | 1 |

n.d: not done

**Table S2.** Characteristics of peptides predicted to bind to HLA-DP4 and selected for binding assays. Affinity ratio was calculated as following: % Rank of wild type - % Rank Mutant / % Rank wild type.

| **Peptide ID** | **Wild Type Peptide** | | **Mutant Peptide** | | **Affinity Ratio** | **Gene** | **aa Substitution** |
| --- | --- | --- | --- | --- | --- | --- | --- |
|  | **aa Sequence** | **% Rank** | **aa Sequence** | **% Rank** |  |  |  |
| **68** | KAFSTCA**S**HLSVVSL | 33 | KAFSTCA**F**HLSVVSL | 0.29 | 0.991212 | OR7A5 | S243F |
| **69** | YKAFSTCA**S**HLSVVS | 26 | YKAFSTCA**F**HLSVVS | 0.24 | 0.990769 | OR7A5 | S243F |
| **70** | QGKYKAFSTCA**S**HLS | 23.12 | QGKYKAFSTCA**F**HLS | 0.26 | 0.988754 | OR7A5 | S243F |
| **71** | KFESEK**H**QDILMWNY | 40.55 | KFESEK**Y**QDILMWNY | 1.88 | 0.953637 | B3GNT2 | H205Y |
| **72** | TCVKFYIEG**S**EPGKQ | 28.72 | TCVKFYIEG**F**EPGKQ | 1.81 | 0.936978 | TIMM21 | S200F |
| **73** | FESEK**H**QDILMWNYR | 45.59 | FESEK**Y**QDILMWNYR | 3.15 | 0.930906 | B3GNT2 | H205Y |
| **74** | HTCVKFYIEG**S**EPGK | 25.25 | HTCVKFYIEG**F**EPGK | 2.04 | 0.919208 | TIMM21 | S200F |
| **75** | CVKFYIEG**S**EPGKQG | 29.58 | CVKFYIEG**F**EPGKQG | 2.52 | 0.914807 | TIMM21 | S200F |
| **77** | HLVA**T**GISCFLGSGL | 38.45 | HLVA**F**GISCFLGSGL | 3.65 | 0.905072 | SEMA5B | T1040F |
| **78** | SFQHLHRLT**C**LKLWY | 23.28 | SFQHLHRLT**Y**LKLWY | 2.38 | 0.897766 | LRRC8A | C643Y |
| **79** | IHLVA**T**GISCFLGSG | 35.22 | IHLVA**F**GISCFLGSG | 3.95 | 0.887848 | SEMA5B | T1040F |
| **80** | FQHLHRLT**C**LKLWYN | 23.73 | FQHLHRLT**Y**LKLWYN | 2.68 | 0.887063 | LRRC8A | C643Y |
| **81** | LVA**T**GISCFLGSGLL | 22.49 | LVA**F**GISCFLGSGLL | 2.68 | 0.880836 | SEMA5B | T1040F |
| **82** | QHLHRLT**C**LKLWYNH | 23.73 | QHLHRLT**Y**LKLWYNH | 3.08 | 0.870206 | LRRC8A | C643Y |
| **83** | IYSLRN**K**EFKSALRR | 34.51 | IYSLRN**T**EFKSALRR | 5.6 | 0.837728 | OR10K1 | K295T |
| **84** | MIYSLRN**K**EFKSALR | 30.49 | MIYSLRN**T**EFKSALR | 5.38 | 0.823549 | OR10K1 | K295T |
| **85** | GYSY**G**ACVAFEMCSQ | 20.46 | GYSY**V**ACVAFEMCSQ | 3.7 | 0.819159 | FASN | G2310V |
| **86** | RNTIGQVAAGAFA**D**L | 33.31 | RNTIGQVAAGAFA**N**L | 6.39 | 0.808166 | LRFN1 | D111N |
| **87** | QPEGPYRVAGYSY**G**A | 64.78 | QPEGPYRVAGYSY**V**A | 12.47 | 0.807502 | FASN | G2310V |
| **88** | SIDRTVMYYGL**P**FIQ | 20.35 | SIDRTVMYYGL**L**FIQ | 3.93 | 0.80688 | SLC3A1 | P400L |
| **89** | CVTE**C**KEDQFRCKNK | 85.41 | CVTE**Y**KEDQFRCKNK | 17.36 | 0.796745 | LRP1B | C3632Y |
| **90** | VTE**C**KEDQFRCKNKA | 85.41 | VTE**Y**KEDQFRCKNKA | 17.45 | 0.795691 | LRP1B | C3632Y |
| **91** | TFRSPQK**V**GALIFLV | 26.8 | TFRSPQK**F**GALIFLV | 5.63 | 0.789925 | SLC38A4 | V513F |
| **92** | PMIYSLRN**K**EFKSAL | 27.31 | PMIYSLRN**T**EFKSAL | 6.05 | 0.778469 | OR10K1 | K295T |
| **93** | GLQTL**D**SAALYHLTT | 23.02 | GLQTL**Y**SAALYHLTT | 5.12 | 0.777585 | LRRC52 | D166Y |
| **94** | CDVPQMLKLAC**S**YEF | 65.11 | CDVPQMLKLAC**Y**YEF | 14.93 | 0.770696 | OR14J1 | S188Y |
| **95** | MDCVTE**C**KEDQFRCK | 84.8 | MDCVTE**Y**KEDQFRCK | 19.74 | 0.767217 | LRP1B | C3632Y |
| **96** | TDLHM**P**GAPVWAMLF | 67.19 | TDLHM**L**GAPVWAMLF | 15.9 | 0.763358 | SLC6A18 | P397L |
| **97** | RNTGLQTL**D**SAALYH | 49.03 | RNTGLQTL**Y**SAALYH | 11.76 | 0.760147 | LRRC52 | D166Y |
| **98** | LLKF**R**EDEGFVREEE | 30.24 | LLKF**W**EDEGFVREEE | 7.32 | 0.757937 | KCNA4 | R270W |
| **99** | IGQVAAGAFA**D**LRAL | 25.43 | IGQVAAGAFA**N**LRAL | 6.39 | 0.748722 | LRFN1 | D111N |
| **100** | LKF**R**EDEGFVREEED | 32.55 | LKF**W**EDEGFVREEED | 8.42 | 0.741321 | KCNA4 | R270W |
| **101** | ALLKF**R**EDEGFVREE | 30.24 | ALLKF**W**EDEGFVREE | 7.91 | 0.738426 | KCNA4 | R270W |

| **102** | TGLQTL**D**SAALYHLT | 20.68 | TGLQTL**Y**SAALYHLT | 5.48 | 0.73501 | LRRC52 | D166Y |
| --- | --- | --- | --- | --- | --- | --- | --- |
| **103** | I**G**KGRFGEVWRGRWR | 52.98 | I**V**KGRFGEVWRGRWR | 14.31 | 0.729898 | ACVR1B | G214V |
| **104** | PRGKGL**G**YAQYLPKE | 23.93 | PRGKGL**V**YAQYLPKE | 6.69 | 0.720435 | AFG3L2 | G604V |
| **105** | GKGL**G**YAQYLPKEQY | 22.44 | GKGL**V**YAQYLPKEQY | 6.36 | 0.716578 | AFG3L2 | G604V |
| **106** | NLSWISIRR**R**QESLG | 51.88 | NLSWISIRR**L**QESLG | 14.93 | 0.712221 | OPN4 | R419L |
| **107** | LGASMVYLI**R**VARRN | 28.6 | LGASMVYLI**L**VARRN | 8.38 | 0.706993 | SPINT2 | R222L |
| **108** | LLSTV**V**RFAVKAIIS | 24.34 | LLSTV**F**RFAVKAIIS | 7.24 | 0.702547 | ABCC9 | V592F |
| **109** | TAFTF**R**SSKEKLDVG | 34.99 | TAFTF**Q**SSKEKLDVG | 18.37 | 0.474993 | RYR2 | R332Q |
| **110** | LQYSG**V**VTWVAMTTQ | 24.55 | LQYSG**M**VTWVAMTTQ | 18.71 | 0.237882 | ADCY8 | V247M |

**Table S3.** Predicted vs actual binding capacity of HLA- DP4-binding neo-epitope candidates. Peptides with IC50 < 200 μM were considered binders.

| **Peptide ID** | **aa Sequence** | **% Rank NetMHC** | **IC50 (nM) NetMHC-II-3.0** | **IC50 (**μ**M)**  **Binding Assay** |
| --- | --- | --- | --- | --- |
| **68** | KAFSTCA**F**HLSVVSL | 0.29 | 119.68 | n.d |
| **69** | YKAFSTCA**F**HLSVVS | 0.24 | 66.38 | 290.7 |
| **70** | QGKYKAFSTCA**F**HLS | 0.26 | 91.11 | 31.5153 |
| **71** | KFESEK**Y**QDILMWNY | 1.88 | 255.28 | 28.94811 |
| **72** | TCVKFYIEG**F**EPGKQ | 1.81 | 101.37 | 261.2 |
| **73** | FESEK**Y**QDILMWNYR | 3.15 | 469 | 15.93939 |
| **74** | HTCVKFYIEG**F**EPGK | 2.04 | 100.47 | 159.1719 |
| **75** | CVKFYIEG**F**EPGKQG | 2.52 | 130.88 | 250.4 |
| **77** | HLVA**F**GISCFLGSGL | 3.65 | 222.22 | 83.96796 |
| **78** | SFQHLHRLT**Y**LKLWY | 2.38 | 106.21 | 25.84841 |
| **79** | IHLVA**F**GISCFLGSG | 3.95 | 271.36 | 397.8 |
| **80** | FQHLHRLT**Y**LKLWYN | 2.68 | 108.32 | 27.76406 |
| **81** | LVA**F**GISCFLGSGLL | 2.68 | 214.99 | 314.3 |
| **82** | QHLHRLT**Y**LKLWYNH | 3.08 | 137.97 | 18.63964 |
| **83** | IYSLRN**T**EFKSALRR | 5.6 | 365.85 | 27.72723 |
| **84** | MIYSLRN**T**EFKSALR | 5.38 | 278.31 | 24.49873 |
| **85** | GYSY**V**ACVAFEMCSQ | 3.7 | 197.89 | 3273 |
| **86** | RNTIGQVAAGAFA**N**L | 6.39 | 2133.02 | 36.95227 |
| **87** | QPEGPYRVAGYSY**V**A | 12.47 | 1767.94 | 31.94385 |
| **88** | SIDRTVMYYGL**L**FIQ | 3.93 | 277.78 | 71.02636 |
| **89** | CVTE**Y**KEDQFRCKNK | 17.36 | 6273.1 | 243.8 |
| **90** | VTE**Y**KEDQFRCKNKA | 17.45 | 6469.83 | 232.1 |
| **91** | TFRSPQK**F**GALIFLV | 5.63 | 105.41 | 24.77032 |
| **92** | PMIYSLRN**T**EFKSAL | 6.05 | 286.13 | 6.35549 |
| **93** | GLQTL**Y**SAALYHLTT | 5.12 | 160.52 | 21.61125 |
| **94** | CDVPQMLKLAC**Y**YEF | 14.93 | 377.08 | 121.7246 |
| **95** | MDCVTE**Y**KEDQFRCK | 19.74 | 6040.57 | 260.1 |
| **96** | TDLHM**L**GAPVWAMLF | 15.9 | 591.88 | 10.87371 |
| **97** | RNTGLQTL**Y**SAALYH | 11.76 | 637.18 | 22.64679 |
| **98** | LLKF**W**EDEGFVREEE | 7.32 | 620.11 | 166.0155 |

| **99** | IGQVAAGAFA**N**LRAL | 6.39 | 952.2 | 27.87704 |
| --- | --- | --- | --- | --- |
| **100** | LKF**W**EDEGFVREEED | 8.42 | 1035.06 | 83.83427 |
| **101** | ALLKF**W**EDEGFVREE | 7.91 | 500.51 | 175.2216 |
| **102** | TGLQTL**Y**SAALYHLT | 5.48 | 160.97 | 14.32093 |
| **103** | I**V**KGRFGEVWRGRWR | 14.31 | 3363.55 | 194.2499 |
| **104** | PRGKGL**V**YAQYLPKE | 6.69 | 296.39 | 38.23184 |
| **105** | GKGL**V**YAQYLPKEQY | 6.36 | 252.66 | 47.91597 |
| **106** | NLSWISIRR**L**QESLG | 14.93 | 734.44 | n.d |
| **107** | LGASMVYLI**L**VARRN | 8.38 | 754.28 | n.d |
| **108** | LLSTV**F**RFAVKAIIS | 7.24 | 307.9 | 2667 |
| **109** | TAFTF**Q**SSKEKLDVG | 18.37 | 3054.49 | 138.3471 |
| **110** | LQYSG**M**VTWVAMTTQ | 18.71 | 1418.88 | 1543 |
| **TT 947-967** | FNNFTVSFWLRVPKVSASHLE | n.d | 19.93 | 3.053931 |

Peptides highlighted in yellow were considered non-binders. TT_947-967_ is a known

HLA-DP4-binder and was used as a positive control in the binding assays. n.d: not determined.

**Table S4.** Binding capacity vs immunogenicity of HLA-DP4-binding neo-epitope candidates.

| **Peptide ID** | **aa Sequence** | **IC50 (**μ**M)**  **Binding Assay** | **Immunogenicity** | |
| --- | --- | --- | --- | --- |
|  |  |  | **No of responses** | **% of responses** |
| **70** | QGKYKAFSTCA**F**HLS | 31.5153 | 7 of 9 | 77 |
| **71** | KFESEK**Y**QDILMWNY | 28.94811 | 3 of 9 | 33 |
| **73** | FESEK**Y**QDILMWNYR | 15.93939 | 2 of 9 | 22 |
| **74** | HTCVKFYIEG**F**EPGK | 159.1719 | 1 of 9 | 11 |
| **77** | HLVA**F**GISCFLGSGL | 83.96796 | 8 of 9 | 88 |
| **78** | SFQHLHRLT**Y**LKLWY | 25.84841 | 3 of 9 | 33 |
| **80** | FQHLHRLT**Y**LKLWYN | 27.76406 | 2 of 9 | 22 |
| **82** | QHLHRLT**Y**LKLWYNH | 18.63964 | 2 of 9 | 22 |
| **83** | IYSLRN**T**EFKSALRR | 27.72723 | 2 of 9 | 22 |
| **84** | MIYSLRN**T**EFKSALR | 24.49873 | 0 of 9 | 0 |
| **86** | RNTIGQVAAGAFA**N**L | 36.95227 | 0 of 9 | 0 |
| **87** | QPEGPYRVAGYSY**V**A | 31.94385 | 1 of 9 | 11 |
| **88** | SIDRTVMYYGL**L**FIQ | 71.02636 | 9 of 9 | 100 |
| **91** | TFRSPQK**F**GALIFLV | 24.77032 | 4 of 9 | 44 |
| **92** | PMIYSLRN**T**EFKSAL | 6.35549 | 2 of 9 | 22 |
| **93** | GLQTL**Y**SAALYHLTT | 21.61125 | 3 of 9 | 33 |
| **94** | CDVPQMLKLAC**Y**YEF | 121.7246 | 0 of 9 | 0 |
| **96** | TDLHM**L**GAPVWAMLF | 10.87371 | 4 of 9 | 44 |
| **97** | RNTGLQTL**Y**SAALYH | 22.64679 | 1 of 9 | 11 |
| **98** | LLKF**W**EDEGFVREEE | 166.0155 | 1 of 9 | 11 |

| **99** | IGQVAAGAFA**N**LRAL | 27.87704 | 4 of 9 | 44 |
| --- | --- | --- | --- | --- |
| **100** | LKF**W**EDEGFVREEED | 83.83427 | 2 of 9 | 22 |
| **101** | ALLKF**W**EDEGFVREE | 175.2216 | 4 of 9 | 44 |
| **102** | TGLQTL**Y**SAALYHLT | 14.32093 | 1 of 9 | 11 |
| **103** | I**V**KGRFGEVWRGRWR | 194.2499 | 2 of 9 | 22 |
| **104** | PRGKGL**V**YAQYLPKE | 38.23184 | 1 of 9 | 11 |
| **105** | GKGL**V**YAQYLPKEQY | 47.91597 | 1 of 9 | 11 |
| **109** | TAFTF**Q**SSKEKLDVG | 138.3471 | 1 of 9 | 11 |

**Table S5. Binding capacity vs immunogenicity of HLA-DP4-binding neo-epitope candidates.** Peptides were grouped according to their binding capacity, based on the IC50 values in cell-based competitive binding assay. The top 10 binders are highlighted in green, the bottom 10, in red and the intermediate, in yellow. Note that two peptides (88 and 77) in the bottom 10 binders were the 2 most immunogenic peptides, while three peptides (102, 97 and 84) in the top 10 were poorly immunogenic.

| **Peptide ID** | **aa Sequence** | **IC50 (**μ**M)**  **Binding Assay** | **Immunogenicity** | |
| --- | --- | --- | --- | --- |
|  |  |  | **No of responses** | **% of responses** |
| **92** | PMIYSLRNTEFKSAL | 6.35549 | 2 of 9 | 22 |
| **96** | TDLHMLGAPVWAMLF | 10.87371 | 4 of 9 | 44 |
| **102** | TGLQTLYSAALYHLT | 14.32093 | 1 of 9 | 11 |
| **73** | FESEKYQDILMWNYR | 15.93939 | 2 of 9 | 22 |
| **82** | QHLHRLTYLKLWYNH | 18.63964 | 2 of 9 | 22 |
| **93** | GLQTLYSAALYHLTT | 21.61125 | 3 of 9 | 33 |
| **97** | RNTGLQTLYSAALYH | 22.64679 | 1 of 9 | 11 |
| **84** | MIYSLRNTEFKSALR | 24.49873 | 0 of 9 | 0 |
| **91** | TFRSPQKFGALIFLV | 24.77032 | 4 of 9 | 44 |
| **78** | SFQHLHRLTYLKLWY | 25.84841 | 3 of 9 | 33 |
| **83** | IYSLRNTEFKSALRR | 27.72723 | 2 of 9 | 22 |

| **80** | FQHLHRLTYLKLWYN | 27.76406 | 2 of 9 | 22 |
| --- | --- | --- | --- | --- |
| **99** | IGQVAAGAFANLRAL | 27.87704 | 4 of 9 | 44 |
| **71** | KFESEKYQDILMWNY | 28.94811 | 3 of 9 | 33 |
| **70** | QGKYKAFSTCAFHLS | 31.5153 | 7 of 9 | 77 |
| **87** | QPEGPYRVAGYSYVA | 31.94385 | 1 of 9 | 11 |
| **86** | RNTIGQVAAGAFANL | 36.95227 | 0 of 9 | 0 |
| **104** | PRGKGLVYAQYLPKE | 38.23184 | 1 of 9 | 11 |
| **105** | GKGLVYAQYLPKEQY | 47.91597 | 1 of 9 | 11 |
| **88** | SIDRTVMYYGLLFIQ | 71.02636 | 9 of 9 | 100 |
| **100** | LKFWEDEGFVREEED | 83.83427 | 2 of 9 | 22 |
| **77** | HLVAFGISCFLGSGL | 83.96796 | 8 of 9 | 88 |
| **94** | CDVPQMLKLACYYEF | 121.7246 | 0 of 9 | 0 |
| **109** | TAFTFQSSKEKLDVG | 138.3471 | 1 of 9 | 11 |
| **74** | HTCVKFYIEGFEPGK | 159.1719 | 1 of 9 | 11 |
| **98** | LLKFWEDEGFVREEE | 166.0155 | 1 of 9 | 11 |
| **101** | ALLKFWEDEGFVREE | 175.2216 | 4 of 9 | 44 |
| **103** | IVKGRFGEVWRGRWR | 194.2499 | 2 of 9 | 22 |

**Table S6.** Characteristics of Capan-1-derived peptides predicted to bind to HLA-DP4 and selected for immunogenicity and binding assays. TPM: Transcripts per million.

| **Peptide ID** | **Gene Name** | **Amino Acid Mutation** | **Aff (nM) NetMHC** | **Gene Expression (TPM)** | **Amino Acid Sequence** |
| --- | --- | --- | --- | --- | --- |
| 1 | RHOT2 | P/L | 20.882 | 36.65 | HAELH**L**SSFWLRGLL |
| 2 | REXO1 | R/W | 37.936 | 7.53 | ECYYHWG**W**LRRNRVA |
| 3 | BCAS1 | V/F | 73.806 | 4.12 | TPGHAPAQDK**F**LSAA |
| 4 | EIF4G2 | R/S | 31.098 | 230.96 | MRSDFFLEGPFMPP**S** |
| 5 | HGS | S/Y | 109.115 | 28.87 | PPEYLTSPL**Y**QQSQL |
| 6 | TMEM185A | A/S | 19.004 | 6.55 | VFMPLFFVSPVSV**S**A |
| 7 | MROH1 | S/Y | 64.909 | 19.05 | VAFC**Y**ALQRFSEGAL |
| 8 | AGAP3 | D/H | 58.568 | 22.99 | EIVVDGQSYLLLIR**H** |
| 9 | IL15 | H/P | 65.074 | 1.83 | CLLLNS**P**FLTEAGIH |
| 10 | CDK20 | S/L | 32.055 | 4.41 | PHGGGFVLAFEFML**L** |
| 11 | FBXW2 | Q/E | 60.059 | 9.73 | LSFYLLKWLDP**E**TLL |
| 12 | TRADD | Q/X | 297.133 | 9.08 | PPPPPAQTFLF**R**VSL |

**Table S7.** Predicted vs actual binding capacity of Capan-1 HLA-DP4-binding neo-epitope candidates.


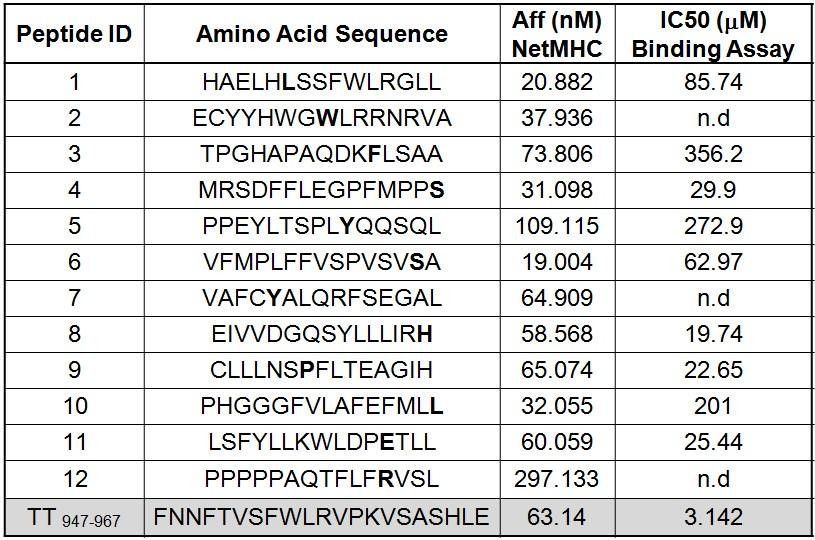


TT_947-967_ is a known HLA-DP4-binder and was used as a positive control in the binding assays. n.d: not determined.
